# Expression and epigenomic landscape of the sex chromosomes in mouse post-meiotic male germ cells

**DOI:** 10.1186/s13072-016-0099-8

**Published:** 2016-10-27

**Authors:** Charlotte Moretti, Daniel Vaiman, Frederic Tores, Julie Cocquet

**Affiliations:** 1 Institut National de la Sante et de la Recherche Medicale (INSERM) U1016, Institut Cochin, Paris, France; 2 Centre National de la Recherche Scientifique (CNRS), UMR8104, Paris, France; 3 Faculté de Médecine, Université Paris Descartes, Sorbonne Paris Cité, Paris, France; 4 INSERM U1163, Université Paris Descartes, Sorbonne Paris Cité, Institut Imagine, 24 Boulevard du Montparnasse, 75015 Paris, France

**Keywords:** Sex chromosomes, Post-meiotic sex chromatin, MSCI, Spermiogenesis, Mouse, H3K4me3, Crotonylation, H3K9me3, H3K27ac, Chromosome 14

## Abstract

**Background:**

During meiosis, the X and Y chromosomes are transcriptionally silenced. The persistence of repressive chromatin marks on the sex chromatin after meiosis initially led to the assumption that XY gene silencing persists to some extent in spermatids. Considering the many reports of XY-linked genes expressed and needed in the post-meiotic phase of mouse spermatogenesis, it is still unclear whether or not the mouse sex chromatin is a repressive or permissive environment, after meiosis.

**Results:**

To determine the transcriptional and chromatin state of the sex chromosomes after meiosis, we re-analyzed ten ChIP-Seq datasets performed on mouse round spermatids and four RNA-seq datasets from male germ cells purified at different stages of spermatogenesis. For this, we used the last version of the genome (mm10/GRCm38) and included reads that map to several genomic locations in order to properly interpret the high proportion of sex chromosome-encoded multicopy genes. Our study shows that coverage of active epigenetic marks H3K4me3 and Kcr is similar on the sex chromosomes and on autosomes. The post-meiotic sex chromatin nevertheless differs from autosomal chromatin in its enrichment in H3K9me3 and its depletion in H3K27me3 and H4 acetylation. We also identified a posttranslational modification, H3K27ac, which specifically accumulates on the Y chromosome. In parallel, we found that the X and Y chromosomes are enriched in genes expressed post-meiotically and display a higher proportion of spermatid-specific genes compared to autosomes. Finally, we observed that portions of chromosome 14 and of the sex chromosomes share specific features, such as enrichment in H3K9me3 and the presence of multicopy genes that are specifically expressed in round spermatids, suggesting that parts of chromosome 14 are under the same evolutionary constraints than the sex chromosomes.

**Conclusions:**

Based on our expression and epigenomic studies, we conclude that, after meiosis, the mouse sex chromosomes are no longer silenced but are nevertheless regulated differently than autosomes and accumulate different chromatin marks. We propose that post-meiotic selective constraints are at the basis of the enrichment of spermatid-specific genes and of the peculiar chromatin composition of the sex chromosomes and of parts of chromosome 14.

**Electronic supplementary material:**

The online version of this article (doi:10.1186/s13072-016-0099-8) contains supplementary material, which is available to authorized users.

## Background

Sex chromosomes differ from autosomes in their genome organization, gene content and gene expression. At the basis of those differences is the fact that they are under different evolutionary constraints due to loss of recombination and sexual antagonism (i.e., when a gene is beneficial to one sex but detrimental to the other) [1–6]. In mammalian males, sex chromosomes are highly heteromorphic and only recombine via a small region usually located at the tip of the chromosome (the PAR, pseudoautosomal region), while in females the X chromosome can recombine. Meaning that, compared to a classic pair of autosomes, the X only recombines two-thirds of the time and most of the Y chromosome does not recombine at all, and is hence called MSY for male-specific region on the Y [7, 8].

One particular phenomenon which affects mammalian sex chromosome gene content and expression takes place in male germ cells: During meiosis, the X and Y chromosomes are transcriptionally silenced by a series of chromatin-based events. The precise role of meiotic sex chromosome inactivation (MSCI) is unknown, but it is conserved and essential in all mammals studied so far and in more distant species such as *C. elegans* (see [9–12] for reviews). While XY chromosomes are enriched in genes expressed in spermatogonia, they are devoid of genes expressed during meiosis, as a consequence of MSCI [13, 14]. Whether or not XY chromosomes remain to some extent silent after meiosis is still controversial (see below).

Based on many studies performed using mostly mouse as a model, MSCI is known to start in spermatocytes at the pachytene stage of meiotic prophase I with phosphorylation of histone H2A variant X by ATR- and MDC1-mediated spreading of this signal over the sex chromosomes [15, 16]. This is followed by changes in histone posttranslational modifications, such as di- and trimethylation of the lysine 9 of histone H3 (H3K9me2 and me3) and ubiquitination of histone H2A (uH2A), and recruitment of heterochromatin proteins (CBX1 and CBX3) [17–21]. Some other changes in the sex chromatin appear later, such as deacetylation of histones H3 and H4 [19], replacement of the canonical histones H3 (H3.1 and H3.2) by H3.3 variant and methylation of the lysine 20 of histone H4 (H4K20me) at mid-pachytene [22]. All these changes in the composition of the sex chromatin are accompanied by its compaction and re-localization at the periphery of the spermatocyte nucleus in a structure called the sex body [23].

**Fig. 1:**
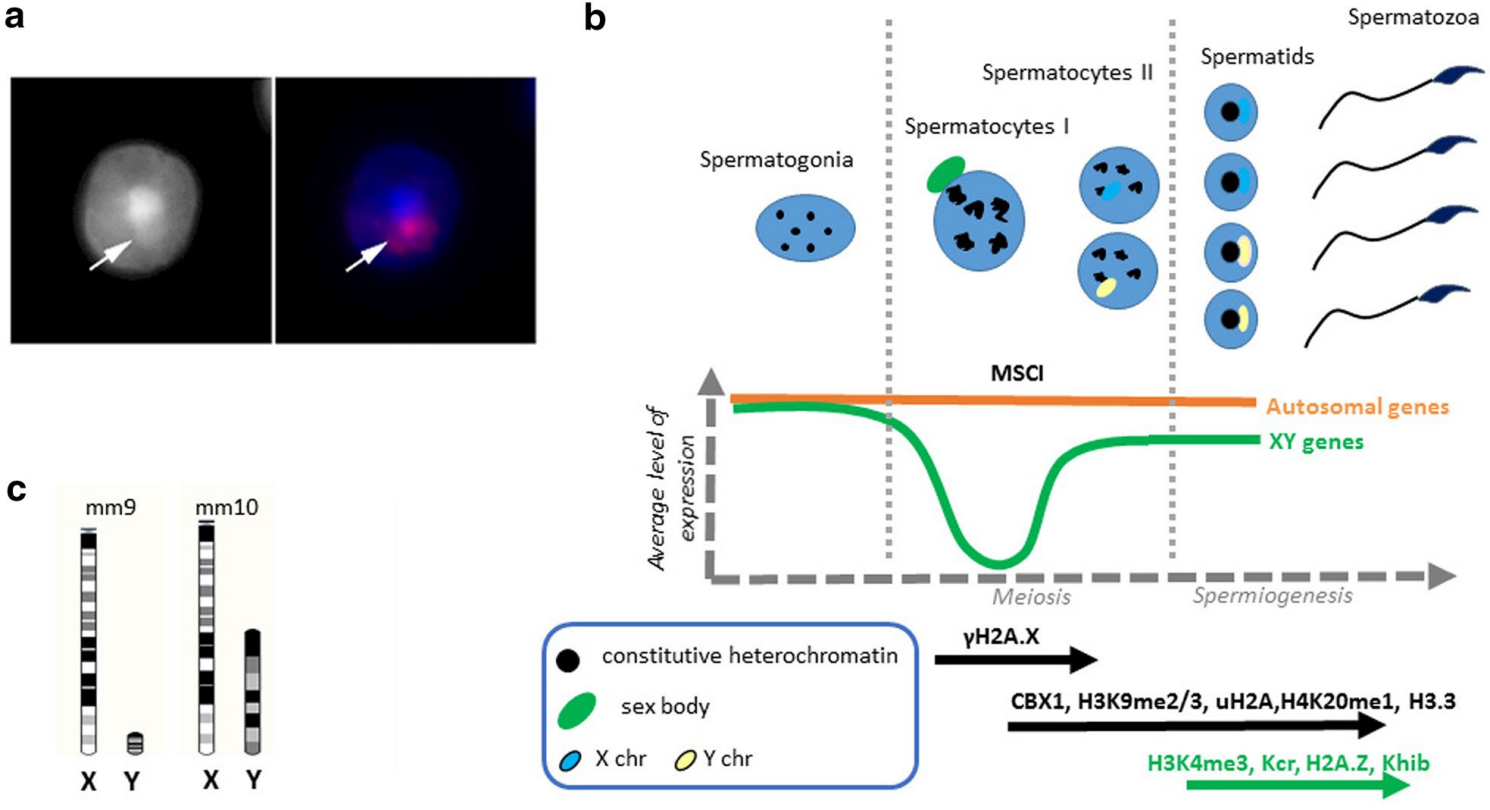
Post-meiotic sex chromatin. **a** Immunofluorescence pictures of a round spermatid nucleus stained with DAPI (*left* in* black* and* white*;* right* in* blue*) and with an X chromosome paint (*pink*). The bright DAPI-dense region is the chromocenter; the* arrow* indicates the adjacent DAPI-dense region in which the X chromosome is located. A similar pattern is observed with a Y chromosome paint [24–26, 34]. **b** Schematic diagram of XY gene expression during mouse spermatogenesis (adapted from [37]). In spermatogonia, X and Y gene expression is comparable to that of autosomes. At the pachytene stage of meiosis I, XY genes are transcriptionally shut down by MSCI (meiotic sex chromosome inactivation). After meiosis, XY gene expression is reactivated. The timing of appearance of the chromatin marks enriched on the sex chromosomes (as observed by immunofluorescence) during and after meiosis is represented under the schematic diagram. **c** Representation of the size of the mouse sex chromosomes in mm9/NCBIM37 (April 2007) and in mm10/GRCm38 (January 2012). In mm9/NCBIM37, the X and Y chromosomes are, respectively, 166.7 and 16 Mb, while in mm10/GRCm38 the X and Y chromosomes are, respectively, 171 and 91.8 Mb. Adapted from Ensembl http://www.ensembl.org/index.html

After meiosis, in spermatids, the sex chromatin (either from the X or the Y chromosome since spermatids are haploid) can still be easily distinguished from autosomal chromatin, as a more DAPI-dense structure immediately adjacent to the constitutive heterochromatin regrouped into one or two chromocenters (Fig. 1a) [24–26]. Some of the repressive chromatin marks and chromatin-associated proteins observed on the sex body during meiosis (H3K9me2 and H3K9me3, CBX1 and CBX3) are still visibly enriched on the post-meiotic sex chromatin [24–26]. These heterochromatin-like features suggest that XY gene silencing persists in spermatids, with most genes repressed and only a few “escapees” [24–28]. There are nevertheless many reports of sex chromosome-encoded genes expressed and needed in the post-meiotic phase of spermatogenesis [29–36] and, in 2008, Mueller et al. have demonstrated that the mouse X chromosome is actually enriched in multicopy genes expressed in spermatids [37].

Interestingly, (re)activation of XY gene expression in spermat**i**ds coincides with changes in nucleosomal histones and histone modifications which appear, by immunofluorescence, to coat the post-meiotic sex chromatin, such as H2A.Z [24], H2A.B3 (also known as H2A.Lap1) [38], histone lysine crotonylation Kcr [39, 40], histone 3 lysine 4 di- and trimethylation (H3K4me2 and H3K4me3) [19, 22], and histone 4 lysine 8 2-hydroxybutyrate (H4K8_hib) [41] (see Fig. 1b). Presence/enrichment of histone variants and histone posttranslational modifications (PTM) on the post-meiotic sex chromatin has been shown to correlate with XY gene expression and, when abnormal, to lead to sperm differentiation defects and male infertility [34, 42–44]. Initially, accumulation of histone variants and PTM onto the post-meiotic sex chromatin was deduced from immunofluorescence observations; but when looked at by chromatin immunoprecipitation followed by high-throughput sequencing (ChIP-Seq), discrepancies with immunofluorescence data were observed. Indeed, by ChIP-Seq, Kcr does not appear to cover more the X chromosome than autosomes [39], and H4K8_hib is even less present on the X chromosome than on autosomes [41]; this is despite the fact that both marks are enriched at the transcriptional start site (TSS) of many post-meiotic activated X-encoded genes (i.e., genes which are upregulated in spermatids compared to spermatocytes) [39, 41]. No conclusion could be drawn for the Y chromosome since, in the mm9 version of the mouse genome available at that time (mm9), only 15% of the Y chromosome was assembled (cf. Fig. 1c).

Therefore, in the present study, we sought to compare the composition of the sex chromatin with that of autosomal using ChIP-Seq and the last version of the mouse genome (mm10/GRCm38). We re-analyzed ten ChIP-Seq datasets performed on mouse round spermatids and showed that active epigenetic marks are similarly present on the sex chromosomes and on autosomes, but that the post-meiotic sex chromatin nevertheless differs from autosomal chromatin in its content in repressive chromatin marks and in histone acetylation. We also identify a histone PTM, H3K27ac, which specifically accumulates on the Y chromosome, suggesting that regulation of the X and Y chromosomes may differ during sperm differentiation.

In parallel, we analyzed RNA-Seq datasets from purified male germ cells to investigate the dynamics of XY gene expression during spermatogenesis, and in particular, after meiosis. We found that the X and Y chromosomes are enriched in genes expressed post-meiotically and display a higher proportion of spermatid-specific genes compared to autosomes though with a range of gene expression values starting lower than autosomal genes. Finally, we found that chromosome 14 and the sex chromosomes share some specific features, such as enrichment in H3K9me3 and the presence of clusters of spermatid-specific genes amplified only in rodents, suggesting that part of chromosome 14 is under the same evolutionary constraints than the sex chromosomes.

Overall, we conclude that, after meiosis, the mouse sex chromosomes are no longer silenced but are nevertheless regulated differently than autosomes and accumulate different chromatin marks. MSCI does not appear to have a strong impact on XY gene expression; we propose that other selective constraints such as the regulatory effect of the X versus Y chromosome intragenomic conflict driven by *Slx/Sly* [45] are at the basis of the significant enrichment of round spermatid-specific genes on the sex chromosomes and on chromosome 14, and of the peculiar features of their chromatin after meiosis.

## Results

### Conserved and specific features of the post-meiotic sex chromatin

There have been several reports of enrichment of histone variants or histone posttranslational modifications on the PMSC based on immunofluorescence observations. This is the case of H2A variants H2A.Z and H2A.B3 (aka H2A.Lap1) [24, 38], H3.3 variant [22], and of histone PTM H3K4me2 [19], H3K4me3 [22], H3K9me2 [19, 22, 25, 26], H3K9me3 [22], H4K16ac [25], histone lysine crotonylation Kcr [39] and histone 4 lysine 8 2-hydroxybutyrate H4K8_hib [41]. ChIP-Seq analyses also highlighted the specific enrichment of Kcr and H2A.B3 at the TSS of a subset of activated X-linked genes after meiosis, as well as the depletion of H4K8_hib on the X chromosome [38, 39, 41].

We sought to determine to what extent sex chromatin differs from autosomal chromatin by systematically re-analyzing 10 ChIP-Seq datasets of histone PTM performed in mouse purified round spermatids using the last version of the mouse genome (mm10/GRCm38). This last version (mm10) is more complete than the previous version (mm9, NCBI built 37) especially regarding Y chromosome sequence (see Fig. 1c) but is still scarcely used in the literature. Importantly, to include multicopy genes in our analysis, reads which mapped perfectly to several genomic regions were not ignored and were distributed to one location picked randomly.

**Fig. 2:**
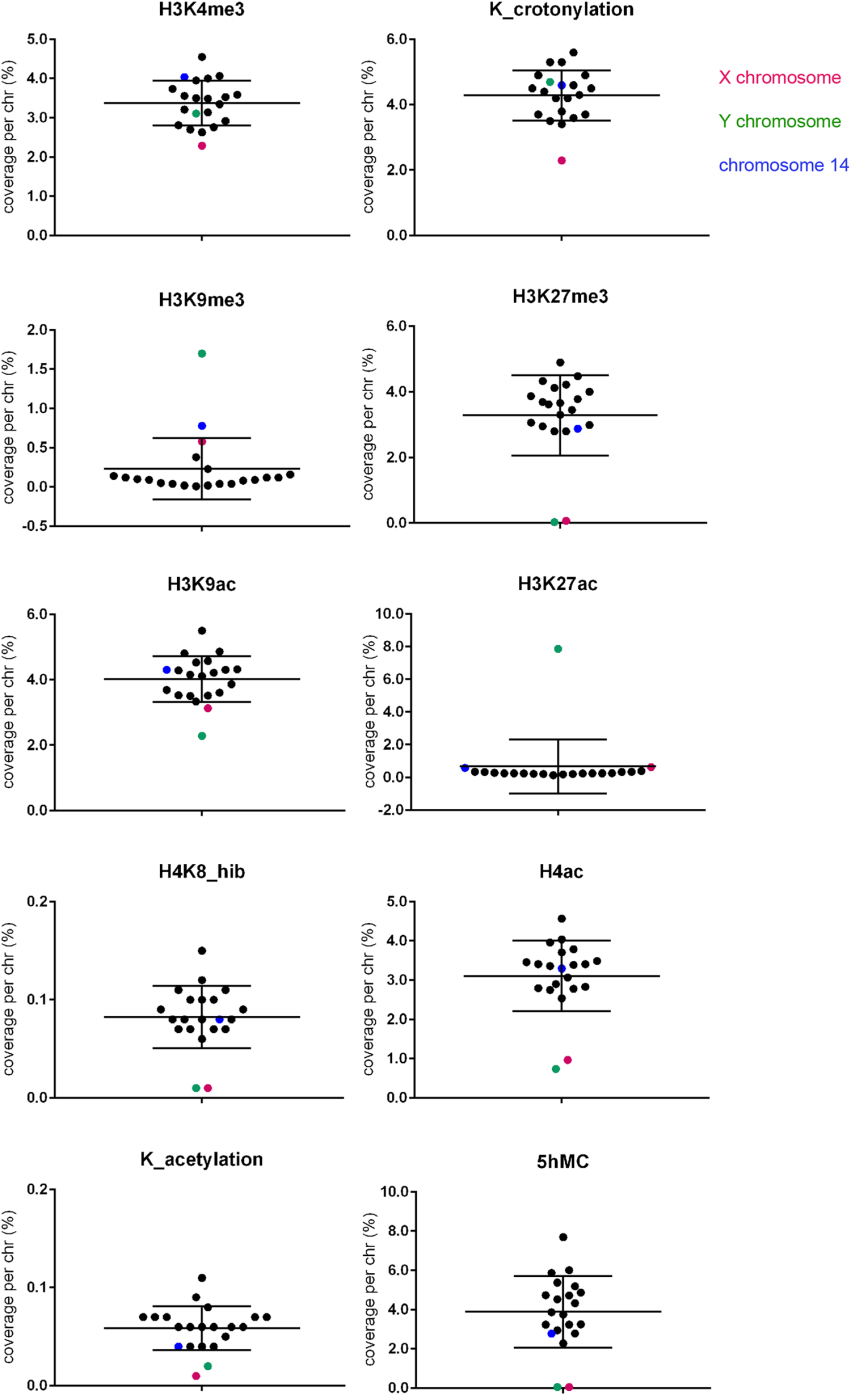
Comparison of the chromatin composition of sex chromosomes and autosomes in mouse round spermatids.* Scatter plot* showing the chromosome coverage (% of each chromosome) of 9 histone PTM (i.e., H3K4me3, Kcr, H3K9ac, H4K8_hib, H4ac, K_acetylation, H3K9me3, H3K27ac, H3K27me3) and 5-hydroxymethylcytosine in round spermatids. Mean values for all chromosomes ± standard deviation are represented. See Additional file 1 for detailed statistical analyses and Additional files 2 and 3 for complementary graphic representations

For each ChIP-Seq dataset, we compared the coverage, i.e., total number of base pairs covered by the chromatin mark, on the X chromosome, the Y chromosome and all autosomes (Fig. 2; Additional file 1). We particularly compared coverage between the sex chromosomes and autosomes of equivalent length and number of genes (i.e., chromosomes 3 and 6 for the X chromosome, and chromosomes 16 and 18 for the Y chromosome, see Table 1 and Additional file 1). Chromosome per chromosome coverage of ChIP peaks was determined in base pair (Additional file 2) and reported to the total length of each chromosome (Fig. 2; Additional file 1). A snapshot of the obtained ChIP-Seq peaks on portion of relevant chromosomes is shown in Additional file 3.

**Table 1:**
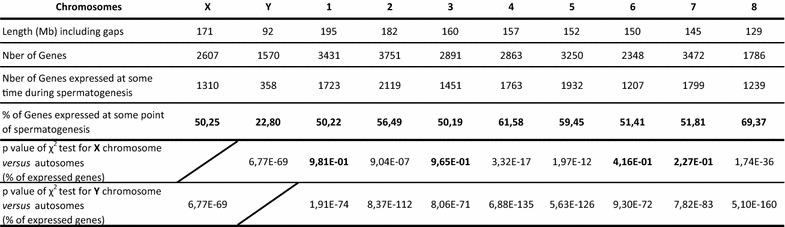
Mouse chromosome statistics (from mm10/GRC38 Ensembl gene 81)

With this method, H3K4me3 and Kcr, two active chromatin marks which appear enriched on the PMSC based on immunofluorescence observations [22, 39, 41], did not have a higher coverage of the sex chromosomes than of autosomes: H3K4me3 and Kcr coverage of the Y was found within the range of that of autosomes, while X chromosome coverage was in fact slightly lower (Fig. 2; Additional files 1, 2 and 3).

With the same approach, we analyzed the repressive chromatin marks H3K9me3 and H3K27me3. Consistent with immunofluorescence observations, H3K9me3 was found enriched on the PMSC compared to autosomes by ChIP-Seq. This enrichment is particularly striking for the Y chromosome since 1.7% of the Y appears covered by H3K9me3 compared to 0.09 and 0.04% of chromosomes 16 and 18, respectively (Fig. 2; Additional file 1). In total, H3K9me3 coverage of the Y chromosome accounts for 25% of its entire genome coverage (while the Y only represents 3.4% of the genome). In comparison, 0.58% of the X is covered by H3K9me3 (compared to 0.1 and 0.04% of chromosomes 3 and 6, respectively) and the X chromosome accounts for 16% of H3K9me3 entire genome coverage (while the X represents 6.3% of the genome). Surprisingly, we observed that chromosome 14 is the second chromosome most enriched in H3K9me3 (0.78% of its length) with H3K9me3 coverage on chromosome 14 accounting for 16% of its entire genome coverage (while chromosome 14 only represents 4.6% of the genome) (Fig. 2; Additional file 1).

As for H3K27me3, it is particularly depleted on both sex chromosomes after meiosis (Fig. 2; Additional files 1 and 2) with 0.07% of the X and 0.03% of the Y chromosome covered by this mark compared to 2.8, 2.95, 3 and 3.3% of chromosomes 3, 6, 16 and 18, respectively (genome coverage: 3.2%) (Fig. 2). The difference in H3K27me3 between the sex chromosomes and autosomes is also very striking when comparing any portion of the sex chromosomes with that of autosomes (Additional file 3). These results are in agreement with previous immunofluorescence observations [22, 25].

**Fig. 3:**
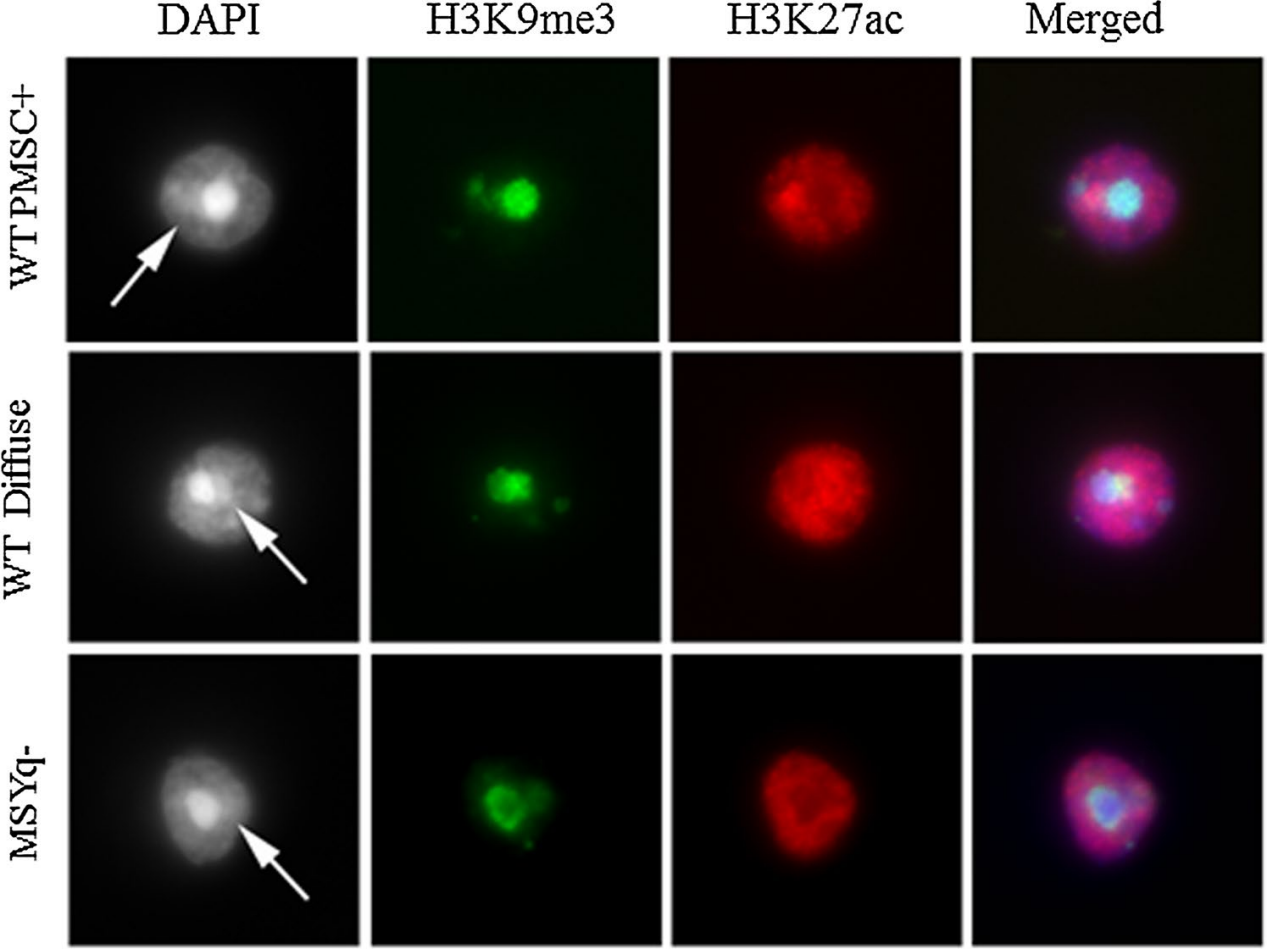
Post-meiotic Y chromosome is enriched in H3K27ac. Immunofluorescence detection of H3K27ac (red) in round spermatid nuclei. DAPI (*blue*) was used to stain nuclei. The most DAPI-dense round region is the chromocenter (i.e., the constitutive pericentromeric heterochromatin) the less DAPI-dense structure adjacent to the chromocenter is the post-meiotic sex chromatin (PMSC) and is indicated by an* arrow*. Anti-H3K9me3 (in* green*) marks the chromocenter and the PMSC in wild type (WT) round spermatids. Two types of staining were observed: either a brighter signal co-localizing with the PMSC (“WT PMSC+” panel), or a diffuse bright signal in the nucleus (“WT Diffuse” panel). As control, round spermatids with a large deletion of the Y chromosome (MSYq-) were used. See Additional file 4 for an extended panel

H3K27me3 and H3K27ac are mutually exclusive; we therefore investigated H3K27ac location in post-meiotic germ cells. Interestingly, we observed an enrichment in H3K27ac on the Y chromosome with 7.9% of the Y covered by this mark compared to 0.3 and 0.2% of chromosomes 16 and 18, respectively (genome coverage: 0.6%). This is not the case of the X chromosome since 0.6% is covered by H3K27ac compared to 0.2 and 0.3% of chromosomes 3 and 6, respectively (Fig. 2; Additional files 1, 2 and 3). To the best of our knowledge, enrichment of H3K27ac on the Y has never been described before; we therefore performed immunofluorescence detection and observed that approximately 50% (28/55) of round spermatids display enrichment of H3K27ac over the PMSC, while the other 50% display a strong diffuse signal not particularly brighter over the PMSC (Fig. 3; Additional file 4). These observations suggested an enrichment of H3K27ac on the Y chromosome (in Y-bearing spermatids which represent ~50% of spermatids) but not on the X (diffuse signal in X-bearing spermatids, which represent ~50% of spermatids). To confirm that, we studied a mouse model with a large deletion (>96%) of its Y chromosome (deletion of the male-specific region of the Y chromosome long arm, MSYq- males) [8, 46]. In this model, the vast majority of round spermatids (59/65) displays a bright diffuse H3K27ac enrichment, without enrichment over the PMSC (Fig. 3; Additional file 4) confirming that H3K27ac enrichment is specific of the Y chromosome.

Looking at other available round spermatid ChIP-Seq datasets for chromatin marks, we found that PMSC is particularly depleted in histone 4 lysine 8 2-hydroxybutyrate (H4K8_hib) in acetylated histone H4 (H4ac) and globally in histone acetylation (K_acetylation) (Fig. 2; Additional files 1, 2 and 3). This confirms previous observations that the X chromosome is depleted in H4K8_hib and H4K8_ac (histone 4 lysine 8 acetylation) in spermatocytes and round spermatids [41]. There is also slightly less H3K9ac on PMSC than on autosomes when normalized to chromosome size (Fig. 2; Additional files 1 and 2).

Finally, we looked at 5-hydroxymethylcytosine (5hMC), a modification at CpG dinucleotides which was previously found less present on the X chromosome in round spermatids [47]; our analyses confirm this observation and show that the Y chromosome is also devoid of 5hMC (Fig. 2; Additional files 1 and 2).

### Dynamics of XY gene expression during spermatogenesis

To compare PMSC features with gene expression profiles, we next re-analyzed RNASeq datasets performed on purified mouse germ cells at different stages of spermatogenesis (spermatogonia B, pachytene spermatocytes, round spermatids and elongating spermatids) [47], using the same mapping parameters we used to analyze ChIP-Seq datasets (i.e., last version of the mouse genome and parameters allowing the mapping of reads from multicopy genes). Using these datasets, we compared the dynamics of expression of sex chromosome-encoded and autosomal genes, according to their RPKM value. The threshold RPKM value (0.22) above which a gene was considered as expressed was calculated by comparing the expression levels of exons and intergenic regions, as previously described in Ramsköld et al. [48]. Importantly, in our analysis we considered one Ensembl gene ID equals one gene, that is to say related genes (such as genes of the same family or copies of the same gene) were not pooled and treated individually.

#### The X chromosome

First, we found that, of the 2607 genes present on the X chromosome, 50.25% (1310 genes) are expressed at least during one stage of spermatogenesis; the same value was obtained for genes on chromosome 3 and chromosome 6 which can be used as reliable comparisons for the X chromosome in term of both length and number of genes (Table 1). It is noteworthy to indicate that for subsequent analysis we took into account genes that are at least expressed in one stage and ignored the genes which are completely silenced during spermatogenesis.

**Fig. 4:**
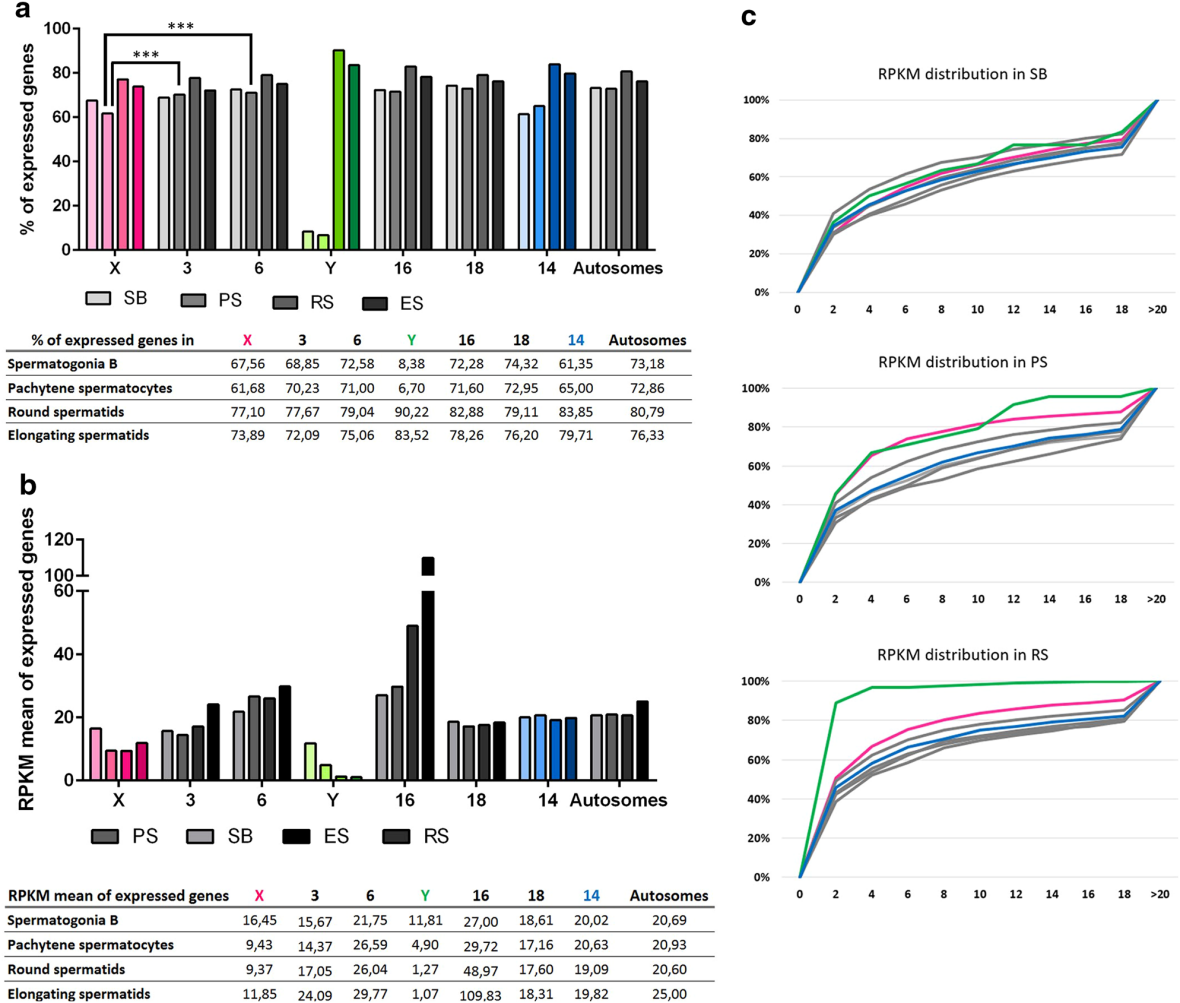
Dynamics of XY gene expression and autosomal gene expression during spermatogenesis. **a** Percentage of expressed genes in spermatogonia B (SB), pachytene spermatocytes (PS), round spermatids (RS) and elongating spermatids for the sex chromosomes (X and Y), five representative chromosomes (chromosomes 3, 6, 14, 16 and 18) and all autosomes. In spermatogonia B, the proportion of expressed genes is similar between the X chromosome and autosomes. At the pachytene stage of meiosis I, the proportion of X-linked expressed genes is decreased due to MSCI. After meiosis, the proportion of expressed genes is similar between the X chromosome and autosomes. The Y chromosome is depleted in genes expressed before meiosis but enriched in round spermatids expressed genes. The percentage values are indicated in the table. The level of statistical significance is marked with three asterisks (*) if p < 0.001. **b** RPKM mean values of expressed genes in spermatogonia B, pachytene spermatocytes, round spermatids and elongating spermatids for the sex chromosomes (X and Y), five representative chromosomes (chromosomes 3, 6, 14, 16 and 18) and all autosomes. The expression level of X- and Y-linked genes is similar to that of autosomal genes in spermatogonia B. The value is indicated in the table. **c** Distribution of RPKM values of expressed genes in spermatogonia B, pachytene spermatocytes and round spermatids. The* X-axis* represents categories of RPKM range of values (0–2, 2–4, 4–6, etc.), the* Y-axis*, the percentage of genes in each category. X-linked genes expression values are indicated in* pink*, Y-linked genes expression values are in green, and autosomal values are in* gray*. After meiosis, the sex chromosomes are enriched in genes with low expression values. See Additional files 5 and 6 for detailed statistical analyses

Just prior to meiosis, in spermatogonia B cells, the proportion of genes expressed varies from approximately 60–70% depending on the chromosome (Fig. 4a). The proportion of expressed X-linked genes is within the range of autosomes, i.e., similar to chromosome 3 but significantly different from chromosome 6 (Fig. 4a; Additional file 5). Similarly, comparison of RPKM values of all genes showed that expression values of X-linked genes are within the range of that of autosomal genes but still with individual differences: this time, the X chromosome values differ from those obtained for chromosome 3 but are similar to chromosome 6 values (Fig. 4b; Additional file 6). These differences reflect the high heterogeneity between autosomes and should be kept in mind when comparing sex chromosomes to autosomes.

**Fig. 5:**
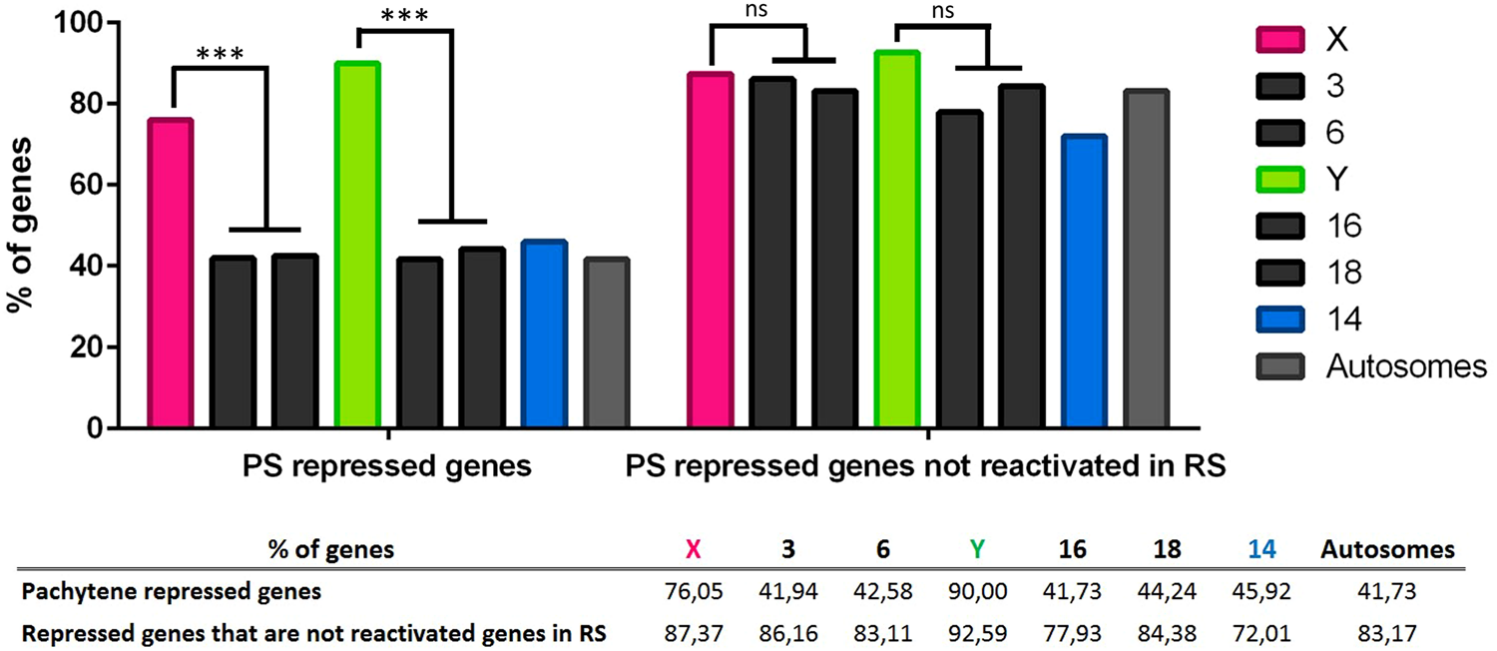
XY and autosomal gene expression in pachytene spermatocytes and in round spermatids. Proportion of pachytene-repressed genes and of non-reactivated genes in round spermatids (RS) for the sex chromosomes (X and Y), five representative chromosomes (chromosomes 3, 6, 14, 16 and 18) and all autosomes. The X chromosome is enriched in pachytene-repressed genes. Similar proportions of non-reactivated genes are found for all chromosomes. Values are indicated in the table. The level of statistical significance is marked with three asterisks (***) if p < 0.001 or with (ns) if not significant. See Additional file 7 for detailed statistical analyses

At the pachytene stage of meiosis, not only a lower proportion of X-linked genes is expressed compared to autosomes (Fig. 4a; Additional file 5) but the RPKM mean value of X-linked genes is also decreased (Fig. 4b; Additional file 6). A higher proportion of pachytene-repressed genes (i.e., expressed before meiosis in type B spermatogonia but no more actively transcribed in pachytene) was also observed among X-linked genes compared to autosomal genes (Fig. 5; Additional file 7), as expected as a consequence of MSCI. However, we still observed many X-encoded genes indicated as “expressed” in pachytene spermatocytes. Those are in fact genes which were expressed before pachytene but of which transcription products are still present in pachytene despite transcription shut down.

**Fig. 6:**
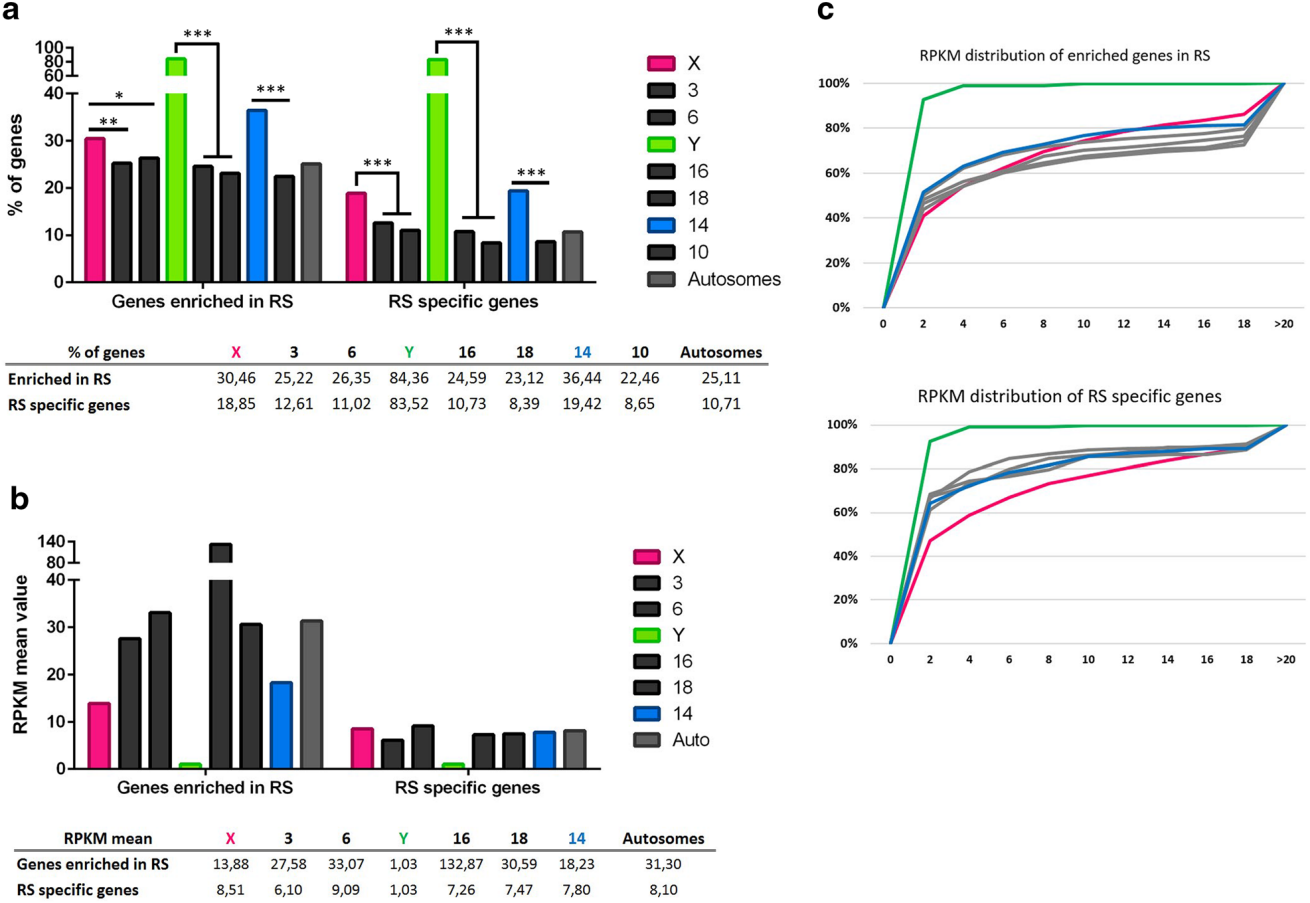
Sex chromosomes and chromosome 14 are enriched in genes expressed after meiosis. **a** Proportion of round spermatid (RS) enriched genes and round spermatid (RS)-specific genes for the sex chromosomes (X and Y), five representative chromosomes (chromosomes 3, 6, 14, 16 and 18) and all autosomes. The proportion of round spermatid-enriched genes and round spermatid-specific genes is significantly higher for the sex chromosomes and chromosome 14 than for the other autosomes. The level of statistical significance is marked with one asterisk (*) if p < 0.05, two (**) if p<0.01.(*) and three (***) if p<0.001. **b** RPKM mean values of round spermatid-enriched genes and specific genes for sex chromosomes (X and Y), five representative chromosomes (chromosomes 3, 6, 14, 16 and 18) and all autosomes. Round spermatid-specific genes encoded by the sex chromosomes display significantly lower values than autosomes. The values are indicated in the table. **c** Distribution of RPKM values of round spermatid-enriched genes and round spermatid-specific genes. The* X-axis* represents categories of RPKM range of values (0–2, 2–4, 4–6, etc.), and the* Y-axis*, the percentage of genes in each category. X-linked genes expression values are indicated in* pink*, Y-linked genes expression values are in* green*, and autosomal values are in* gray*. See Additional files 8 and 9 for detailed statistical analyses

After meiosis, in round spermatids, the majority of pachytene-repressed genes do not recover their prior level of expression and were considered as round spermatids non-reactivated genes (Fig. 5). This is true for autosomal genes and X-encoded genes (Fig. 5; Additional file 7). Of note, an identical proportion of X-linked genes than of autosomal genes are expressed in round spermatids (Fig. 4a; Additional file 5). Surprisingly, 30.46% of X-encoded expressed genes are enriched in round spermatids (i.e., expressed higher in round spermatids than in pachytene spermatocytes). This is, overall, significantly higher than for autosomal genes (25.22 and 26.35% for chromosomes 3 and 6, respectively) (Fig. 6; Additional file 8). Finally, we found that ~18% of X-encoded expressed genes are specifically expressed post-meiotically. This proportion of genes is also significantly higher for the X chromosome than for autosomes (12.61 and 11.02% for chromosomes 3 and 6, respectively) (Fig. 6; Additional file 8). In order to determine whether the high proportion of X-encoded round spermatid-specific genes is due to the abundance of X-linked multicopy genes and to the fact that, in our analysis, we considered one Ensembl gene ID equals one gene, we undertook an additional analysis in which highly similar genes (i.e., genes of the same “family”) were treated as one gene (Additional file 9). The results obtained did not significantly differ from the initial analysis meaning that the chromosome X is genuinely enriched in single-copy genes and multicopy genes specifically expressed at the round spermatids stage (Additional file 9).

Regarding the expression intensity (RPKM) of the genes enriched in round spermatids, the mean value is lower for X-encoded genes than for autosomal genes (chr 3, *m* = 27.58; chr 6, *m* = 33.07; X chr, *m* = 13.88), but unexpectedly it is not significantly different (Fig. 6b, c; Additional file 10). Chromosome 16 displays the highest RPKM value (*m* = 132.87), an odd characteristic explained by the presence of genes massively expressed after meiosis: *Tnp1*, *Prm1* and *Prm2* (which, respectively, encode transition protein 1, protamines 1 and 2). Indeed, their expression value in round spermatids is over 5700 RPKM. Finally, although RPKM means of round spermatid-specific genes are in the same range for the X chromosome and autosomes (from 7.26 to 9.09), X-linked genes display a distribution of RPKM values that is significantly different from that of autosomal genes (Fig. 6c; Additional file 10).

#### The Y chromosome

When focusing on the Y chromosome, we found a very different profile than that of the X chromosome. First, of the 1570 Y-linked genes, only ~23% are expressed at some point during spermatogenesis, compared to 62.69% for chromosome 16 and 65.77% for chromosome 18 (Table 1), which can be used as reliable comparisons for the Y chromosome in term of both length and number of genes. The fact that only 23% of Y chromosome genes were found expressed at some point during spermatogenesis is due to the presence of non-transcribed genes (or with a level of transcription too low to be detected) and non-transcribed pseudogenes belonging to *Sly*, *Ssty1*, Ssty2 or *Srsy* gene family, located on the Y chromosome long arm. Indeed, the proportion of Y-linked genes expressed during spermatogenesis was dramatically increased when only genes (and not pseudogenes) were analyzed (based on the data reported by Soh et al. [8]), and highly similar gene family members of the Y chromosome were treated as a single gene ID (see Additional file 9).

In spermatogonia B cells, only 30 Y-linked genes are expressed; this represents 8.38% of all Y-encoded expressed genes compared to 72.28% of chromosome 16 and 74.32% of chromosome 18 expressed genes (Fig. 4a; Additional file 5). Its RPKM mean value is, however, not significantly different from the corresponding autosomal values (Fig. 4b; Additional file 6). This result can be explained in light of the RPKM value distributions which are overall similar for Y-linked genes and autosomal genes (Fig. 4c; Additional file 6).

At pachytene stage, most of the spermatogonia expressed genes are repressed or completely shut down: 7.54% of expressed genes are pachytene-repressed genes, which means that 90% of the spermatogonial B expressed genes are repressed at the pachytene stage of meiosis. This proportion is not comparable in chromosome 16 and chromosome 18 (Fig. 5; Additional file 7). As a consequence, the proportion of Y-encoded genes “expressed” at this stage drops to 6.7% (Fig. 4a; Additional file 5); their RPKM mean value drops down to 4.90 and is significantly lower than that of autosomes (Fig. 4b; Additional file 5). Strikingly, 90% of Y-linked expressed genes are in fact expressed after meiosis (Fig. 4a), the vast majority of them (83.52%) being exclusively *de novo* expressed in round spermatids (genes first expressed at the spermatid stage) (Fig. 6a) and still found expressed in elongating spermatids (Fig. 4a). These values are significantly higher for the Y than for autosomes (Figs. 4a, 6a; Additional files 5 and 8). The extremely low level of their RPKM values is a peculiarity of Y-linked genes expressed in round spermatids (Figs. 4c, 6c; Additional files 6 and 10).

#### Chromosome 14

**Fig. 7:**
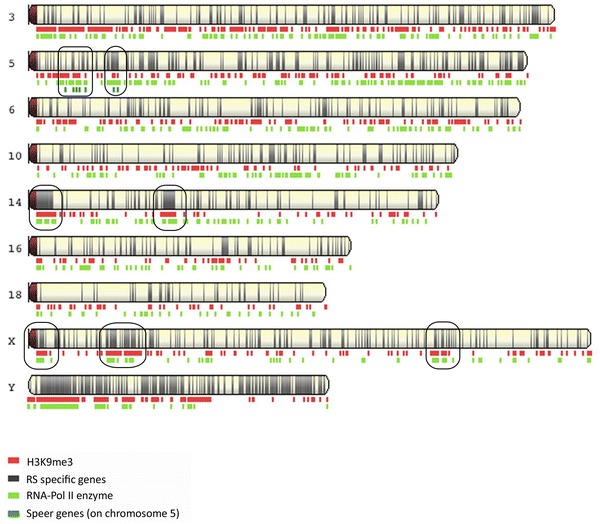
Sex chromosomes and chromosome 14 have common chromatin features in round spermatids. Graphic representation of the ChIP-Seq profiles of H3K9me3 and RNA-Pol II enzyme over chromosomes 3, 5, 6, 10, 14, 16, 18, X and Y in round spermatids. RNA-Pol II (in* green*) marks the regions where round spermatid-expressed genes are located. The genomic location of round spermatid-specific genes is represented as* narrow black bars* on the chromosomes. The genomic location of *Speer* genes is indicated by* blue bars* underneath the chromosomes. Ampliconic multicopy spermatid-specific genes on portions of chromosomes 5, 14 and X are* circled*. Note that they are enriched in H3K9me3 (in* red*). The figure was made using NCBI Genome Decoration Page Web site (using mm10/GRC38 Ensembl gene 81)

**Fig. 8:**
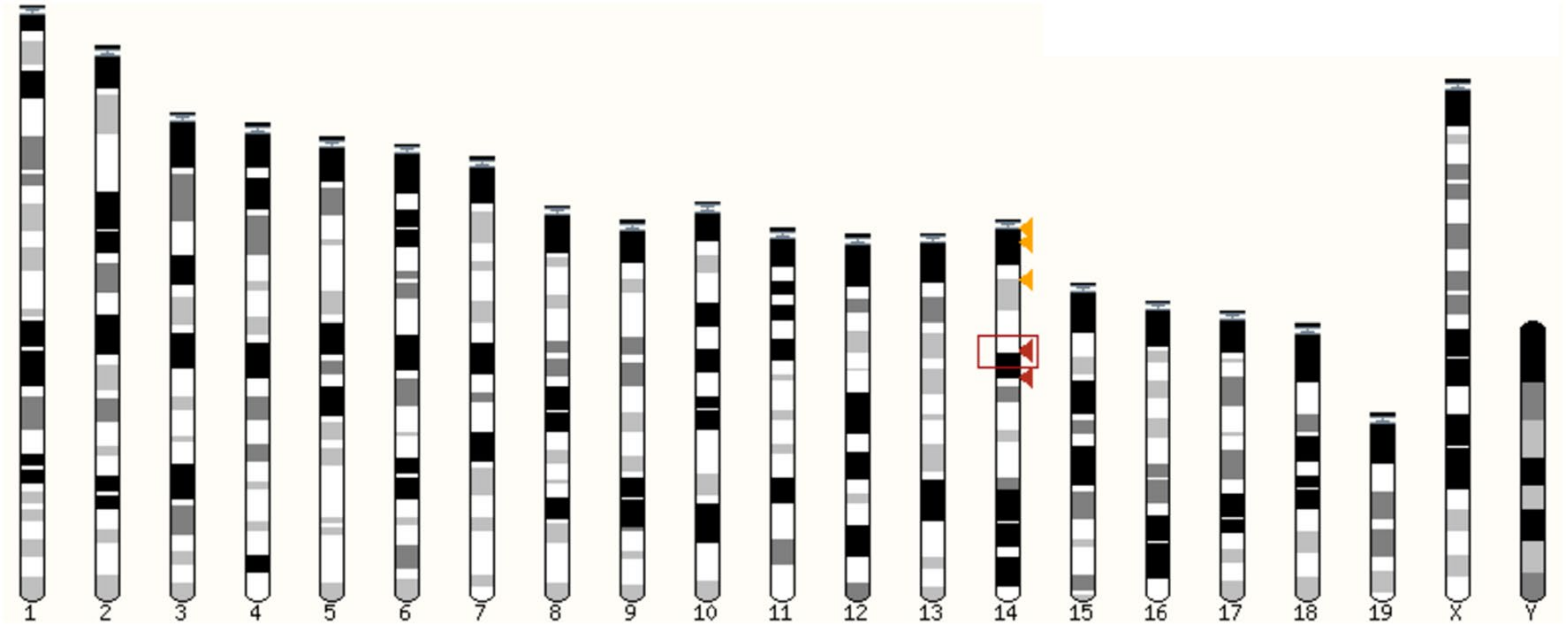
Mouse chromosome 14 encodes many spermatid-specific genes belonging to *α*-*takusan* family and located in two main regions. Graphic view of the chromosomal location of the 277 genes related to *α*-*takusan* family found using Ensembl BLAST/BLAT. Those genes are located in clusters at the tip of the chromosome [chr14: 3,000,000–7,600,000 (indicated by two *orange arrowheads*) and chr14: 19,000,000–19,000,000 (indicated by one *yellow arrowhead*)] or more distal [chr14: 41,200,000–51,800,000 (indicated by two* red arrowheads*)]. The figure was made using Ensembl BLAST/BLAT Web site (using mm10/GRC38 Ensembl gene 81)

Chromosome 14 stands out compared to other autosomes (more particularly to chromosome 10 which can be used as a reliable comparison for chromosome 14 in term of both length and number of genes) and resembles that of the X in its high proportion in spermatid-enriched genes (~36.44%) and in spermatid-specific genes (~19.42%, Fig. 6a; Additional file 8). The mapping of these spermatid-specific genes revealed two regions that are both deprived of round spermatid non-reactivated genes and densely composed of round spermatid-specific genes (Fig. 7; genes listed in Additional file 11). The RPKM mean value of this group of genes is incidentally lower than that of spermatid-specific autosomal genes (3.61 compared to 7.80). Those genes belong to the α-takusan family which has been massively amplified in two regions of mouse chromosome 14 (Fig. 8).

## Discussion

Based on immunofluorescence data of many studies [19, 22, 24–26, 38, 39, 41], the post-meiotic sex chromatin forms a peculiar structure which appears to accumulate more active and repressive histone marks than autosomes. These marks are crucial for the regulation of XY gene expression after meiosis and for normal sperm differentiation; changes in the composition of sex chromatin are associated with deregulation of XY genes, abnormal sperm differentiation and male infertility [34, 42, 43]. Here, we compared the post-meiotic sex chromatin with autosomal chromatin using ChIP-Seq analyses. In parallel, we studied the dynamics of XY gene expression during spermatogenesis using RNA-Seq datasets from purified mouse germ cells.

### The presence of active marks on the PMSC correlates with expression of many XY-encoded genes post-meiotically

First, we found that, following the global transcriptional shut down of XY gene expression during meiosis, chromatin marks associated with open chromatin and gene expression (i.e., H3K4me3 and Kcr) cover the sex chromosomes in the same way as they cover autosomes. This contrasts with immunofluorescence data in which H3K4me3 and Kcr signals appear more intense on the PMSC than on the rest of the nucleus [22, 39, 40] (and personal observations). A similar discrepancy has previously been reported by Dai and colleagues for H4K8_hib and H4K8_ac; indeed, these marks appear enriched on the post-meiotic X and Y chromosomes by immunofluorescence, but their coverage of the X chromosome (obtained by ChIP-Seq) is poor compared to that of autosomes [41]. The authors hypothesized that the X chromosome regions covered by these marks could be particularly accessible to antibodies. Discrepancy between IF and ChIP-Seq may also be explained by the fact that sex chromatin is denser than autosomal chromatin (as suggested by DAPI staining); therefore, sex chromatin immunofluorescence signal accumulates in a smaller, denser region than for autosomes, and appears to be brighter. A similar artifact has been unraveled by Perche and colleagues, who have demonstrated that macro-H2A enrichment on the inactive X of female somatic cells was in fact due to a higher density in nucleosomes [49]. One should nevertheless keep in mind that, in contrary to immunofluorescence experiments, ChIP-Seq analyses are performed on a pool of round spermatids from step 1 to step 8 and may mask/average differences between spermatids at different steps of spermiogenesis. Besides, it is worth noting that independently of the width of their genome coverage, active chromatin marks such as H3K4me3, H2A.B3, Kcr and H4K8_hib are enriched at the TSS of X-encoded and autosomal genes expressed post-meiotically [38, 39, 41] (and personal observations).

Since the sex chromosomes appear to accumulate active chromatin marks in the same way as autosomes after meiosis, we investigated expression of XY genes at that stage. Several studies have concluded that the X chromosome is still under the influence of MSCI after meiosis since they observed that only a small number of genes are expressed from the X chromosome in post-meiotic cells [25, 50, 51]. These studies were based on microarray analyses and either ignored multicopy genes or considered all genes of the same family (all copies of the same gene) as one gene. Here, using parameters allowing interpretation of multicopy genes, we found that the X chromosome is not depleted in genes expressed post-meiotically. *Au contraire*, a high number of X genes are expressed after meiosis; the X chromosome even encompasses a higher proportion of genes which are upregulated in round spermatids than autosomes. Also a higher proportion of genes with spermatid-specific expression (*de novo* expressed genes, i.e., first expressed at the spermatid stage) was found on the X than on autosomes. Our data are in line with results obtained by [37] which showed that the mouse X chromosome encodes 273 genes (which belongs to 33 multicopy gene families) with predominant expression after meiosis.

To the best of our knowledge, no thorough description of the dynamics of mouse Y gene expression during spermatogenesis has been performed so far. This is probably due to the peculiar structure of the mouse Y chromosome (composed of ampliconic euchromatin for >95% of its sequence [8]) and to the fact that most studies used the previous version of the mouse genome (mm9) which is incomplete in terms of Y chromosome sequence. Here, we found that the vast majority of Y-encoded expressed genes are exclusively expressed after meiosis (~83% of expressed genes compared to ~11% for chromosome 16), but with very low expression values. This reflects the presence of 2 gene families (*Sly* and *Ssty*) representing more than 400 genes in total [8], and which are exclusively expressed in spermatids [52, 53].

It is important to note that the threshold value above which a gene is considered as expressed is a critical parameter. For the present study, this value was calculated using a method comparing expression levels of exons and intergenic regions, previously described in Ramsköld et al. [48], and which is lower than the one chosen in other studies such as in [54]. In spite of that, many Y- and X-encoded ampliconic genes which belong to families of genes specifically expressed after meiosis had expression values below that threshold and were considered as silent (~2/3rd of all Y-linked genes). Since the conservation between ampliconic genes of the same family sometimes reaches 99%, it is impossible to precisely determine which gene members are transcribed and which are not. Soh et al. have estimated transcription of Y ampliconic gene families in whole testicular RNA-Seq datasets based on the search of sequence variants that distinguish individual members of each gene family. Out of the 432 genes and 366 pseudogenes of *Sly*/*Ssty* families they could infer, they found evidence of transcription of at least 25% of them (*n* = 208). Transcription of *Srsy* genes was too low to be confidently confirmed [8]. These results are in agreement with those found in our analysis, since we found ~300 Y-encoded genes which are specifically expressed after meiosis.

### Specific features of the PMSC

ChIP-Seq analyses revealed that the sex chromatin bears a signature different from that of autosomal chromatin, characterized by enrichment in H3K9me3 and depletion in histone H4 acetylation, H3K27me3 and 5-hydroxymethylcytosine. These results are in agreement with previous studies in which H3K9me3 enrichment and H3K27me3 depletion have been observed on the sex chromatin during and after meiosis, by immunofluorescence [19, 22, 25].

Repressive chromatin marks H3K9me3 and H3K27me3 are considered to be mutually exclusive [55]. H3K27me3 is usually present at gene-rich regions, while H3K9me3 has been shown to localize to regions with tandem repeats (i.e., centromeres, telomeres and long terminal repeats) but is also found at unique sites, near satellite repeats or long terminal repeats [56]. Here, we found H3K9me3 is more present on the sex chromosomes than on autosomes (with H3K9me3 coverage of the sex chromosomes representing ~40% of H3K9me3 entire genome coverage) and, in comparison, covers more of the Y than of the X chromosome. Despite its highly ampliconic structure, the mouse Y content is similar in interspersed-repeat to mouse autosomes and is rather gene dense. Since more than 95% of its sequence is made of ampliconic euchromatin [8], heterochromatinization of the Y cannot account for H3K9me3 enrichment. A lysine residue cannot be both methylated and acetylated; yet, no major depletion in H3K9ac was observed on the post-meiotic sex chromatin, but this is not surprising since H3K9ac is located at the transcription start sites (TSS) of active genes, while H3K9me3 is reduced at TSS and maps to intergenic regions [57, 58].

Analysis of ChIP-Seq data also revealed that PMSC is depleted in acetylated histone H4 and in acetylated histone globally. Depletion in acetylated histone H4 had been observed on sex chromosomes during meiosis [19] and, recently, Goudarzi et al. have reported the depletion of the X chromosome in acetylated and butyrylated histone H4 residues during and after meiosis [59]. Since two recent studies have shown that BET bromodomain proteins (such as BRDT and BRD4) which are involved in transcriptional activation and chromatin remodeling during spermatogenesis, can bind to acetylated histone H4 lysine residues but not to butyrylated or crotonylated residues [59, 60], it is clear that the expression of sex chromosome-encoded genes is controlled (at least in part) by distinct pathway(s) than that of autosomal genes, in post-meiotic cells. Besides, histone H4 hyper acetylation and BET bromodomain proteins have been shown to be essential for the chromatin remodeling events which ultimately lead to the replacement of histones by protamines during spermatid elongation [61]. Histone H4 hyper acetylation starts a few days after the round spermatid stage, in elongating spermatids. To date, this process has only been studied globally, but it would be interesting to investigate whether the dynamics of histone acetylation and removal in elongating spermatids is different for the sex chromosomes compared to other chromosomes.

Finally, genome mapping of 5-hydroxymethylcytosine had previously shown that this DNA modification is less present on the post-meiotic X chromosome than on autosomes [47]; our study confirmed this observation and showed that the Y chromosome is also devoid of 5hMC.

### Distinct feature between the X and Y chromosomes

H3K27 residue can either be acetylated or methylated, and both modifications are enriched at the TSS. Our analyses showed that the Y chromosome, while devoid of H3K27me3, is enriched in H3K27ac. Since H3K27ac marks the TSS and enhancers of active genes [62], enrichment of H3K27ac on the Y may be correlated with the fact that many Y chromosome-encoded genes are expressed post-meiotically. Despite the presence of many spermatid-expressed genes on the X chromosome, we did not observe a particular enrichment of H3K27ac compared to autosomes suggesting that X and Y chromosome gene regulation may differ. The X and Y chromosomes, while sharing specific features, bear significant differences and may be considered individually rather than as a whole. Using a similar approach, it would be interesting to look at the X and Y chromatin composition in other cell types, germinal and somatic, as well as in other organisms.

### Dynamic of XY gene expression during spermatogenesis

Our analysis provides a comparison of XY gene expression and autosomal gene expression throughout spermatogenesis. Based on those data, we can conclude that, before meiosis, a similar proportion of genes is expressed from the X chromosome and from autosomes, while only a few (30) Y genes are expressed. At the pachytene stage of meiosis, as a consequence of MSCI, a large majority of X and Y genes (76.05 and 90%) can be considered as repressed (i.e., at least twofold downregulated in pachytene spermatocytes compared to spermatogonial B cells), compared to ~40% of autosomal genes.

Interestingly, most genes (whether autosomal or sex chromosome-encoded) which are expressed in spermatogonia and repressed in spermatocytes are not “reactivated” after meiosis, suggesting very distinct genetic programs between the spermatogonia stage and the meiotic/post-meiotic phase. This is in agreement with results obtained in recent transcriptomic studies which described a switch in global gene expression at the initiation of pachynema [50, 54]. After meiosis, despite the fact that the transcription of some genes with spermatid-specific function starts in spermatocytes [54], there is still a substantial number of genes of which expression is enriched in spermatids or even spermatid specific; the sex chromosomes and chromosome 14 are particularly enriched in those genes compared to autosomes.

### Portions of chromosome 14 may be under similar evolutionary constraints than the sex chromosomes

In our study, we observed that chromosome 14 stands out compared to other autosomes and resembles the X chromosome in both its epigenomic features and its high proportion of spermatid-specific genes with lower than autosomal RPKM values. Interestingly, the mapping of these spermatid-specific genes revealed two large genomic regions which encompass ampliconic genes of the α-takusan family. The *α*-*takusan* family (takusan, meaning “many” in Japanese) has been shown to encompass at least 75 genes, and its expression is restrained to brain and testis [63]. We show here that, in the germline, the *α*-*takusan* genes are specifically expressed after meiosis, are enriched in H3K9me3 and have been massively amplified in the mouse lineage, with ~277 genes found in total. Those are features shared with the sex chromosome-encoded genes, *Slx/Sly* and *Spin/Ssty* gene families [32, 35, 53, 64] which have been shown to be involved in the X versus Y chromosome post-meiotic intragenomic conflict (also known as sex chromosome drive). At the basis of this phenomenon is the presence of (a) selfish gene(s) (or transmission distorter) on the X chromosome which favors its own transmission when not balanced. *Slx* and *Sly* have been shown to be regulators of this process via their opposite effects on gene expression [45]. The involvement of *α*-*takusan* genes in this phenomenon remains to be determined, but our results suggest that they are under similar evolutionary constraints than *Slx/Sly* and *Spin/Ssty* gene families. X chromosome drivers are thought to prompt the development of a resistant Y chromosome and of autosomal suppressors (see [65] for a recent review). It is therefore tempting to speculate that the *α*-*takusan* gene family could act as a potential autosomal suppressor of the intragenomic conflict between *Slx* and *Sly* gene families. Investigations on the *α*-*takusan* genes during spermiogenesis will surely shed light on the mechanism(s) underlying this intragenomic conflict.

### Is H3K9me3 enrichment on the PMSC a consequence of MSCI or of the competition between the sex chromosomes?

It is intriguing that portions of chromosome 14 which a priori are not submitted to MSCI are enriched in H3K9me3. In that respect, *α*-*takusan* genes are probably not the only autosomal genes with this chromatin feature associated with gene amplification, but maybe the most striking example of autosomal gene amplification. As such, the *Speer* gene family located on chromosome 5 is another example of autosomal ampliconic spermatid-specific genes [45, 66, 67] but less numerous than *α*-*takusan* genes; it also appears enriched in H3K9me3 (Fig. 7). This might represent the first evidence that H3K9me3 enrichment on the PMSC is due to the post-meiotic intragenomic conflict rather than to MSCI. To address this question, it will be required to determine whether H3K9me3 enrichment on gene-rich portions of chromosome 14 and chromosome 5 is established in spermatocytes or in spermatids.

Whether or not H3K9me3 enrichment is initiated at meiosis or later, it does not prevent XY gene expression in spermatids. Our data suggest that the presence of active chromatin marks, such as H3K4me3, Kcr, H3K9ac and, particularly for the Y, H3K27ac at the TSS of the genes located in an H3K9me3-rich repressive environment is sufficient to ensure transcription by the RNA-Pol II enzyme (Fig. 7).

## Conclusion

In mouse post-meiotic cells, the composition of sex chromatin significantly differs from that of autosomes, such as its enrichment in H3K9me3. The sex chromosomes also contain a higher proportion of genes expressed at their highest after meiosis, though often with lower expression values than autosomal genes. We propose that these features are consequences of the post-meiotic X versus Y intragenomic conflict rather than a consequence of a repressive chromatin environment inherited from the meiotic silencing of sex chromosomes. In other species than the mouse, such as in the cattle and domestic pig [7, 68, 69] sex chromosomes encode clusters of ampliconic genes, it would therefore be interesting to investigate their PMSC composition and dynamics of gene expression during spermatogenesis.

## Methods

### ChIP-Seq analyses

Round spermatids ChIP-Seq datasets from [39, 41, 57, 70, 71] were analyzed as follows. Only those with a high number of reads and a good FastQC report were considered. All samples and input were single end and were aligned using Heng Li Burros-Wheeler aligner BWA [72] version 0.7.5a-r405 with “mem” command against last mouse genome release mm10 (see Additional file 12). By default with BWA, reads which do not map uniquely on the genome are distributed to one location picked randomly. Briefly, BWA-MEM algorithm works by seeding alignments with maximal exact matches (MEMs) and then extending seeds with the affine-gap Smith-Waterman algorithm (SW). In case of multiple primary alignments, -c INT discards a MEM if it has more than INT occurrence in the genome. The maximum is set to 10,000. Peaks were then called with MACS [73] v1.4 using default parameters (p value cut off for peak detection = 1e^−05^; mfold parameters = 10.32), cf. Additional file 12. Decreasing the mfold parameters values did not change the interpretation of ChIP-Seq data. Analyses were partly performed using Mississippi Galaxy Web site https://mississippi.snv.jussieu.fr/.

Integrative Genomic Viewer (IGV) was used to produce graphic representations of H3K9me3, H3K27ac, H3K27me3, H4ac, H3K4me3 and Kcr ChIP peaks on chromosomes X, Y, 3 and 18 [74]. Chromosomes 3 and 18 were used as “representative autosomes” because they have approximately the same size as chromosomes X and Y, respectively. Comparison with other autosomes gave similar results.

### Expression analyses

RNA-Seq datasets from [47] (GSE35005) were analyzed by GenoSplice (http://www.genosplice.com) using the following parameters: Reads were aligned using STAR 2.4.2a [75] on Mouse Genome version GRCm38 (see also Additional file 12). All genes (including pseudogenes) were taken into account. Gene counts were computed using featureCounts from the Subread package version 1.4.6-p5 [76]. RPKM values were computed from Subread results. Ensembl 81 was used for gene annotation. Reads which mapped at different locations were assigned to the location with the best score or to one location picked randomly by the program (option-M, see line commands in Additional file 12). The limit is set to 10 (i.e., reads which mapped to 10 or more locations with a similar score were excluded; this represents less than 0.5% of all reads, cf. Additional file 12). The threshold RPKM value above which a gene was considered as expressed was calculated by comparing the expression levels of exons and intergenic regions (i.e., intersection of density plots), as previously described in Ramsköld et al, see Additional file 13 [48]. Individual threshold RPKM values vary among datasets from 0.15 to 0.27, yielding to an average threshold RPKM value of 0.22. Genes de novo expressed are genes found to be expressed (RPKM > 0.22) at one particular stage (pachytene stage or in round spermatids) and not before. Pachytene-repressed genes are genes that are at least twofold downregulated compared to their RPKM value in spermatogonial B cells. Round spermatids non-reactivated genes are genes defined as repressed in pachytene stage (see above) which do not recover at least half of their spermagonia B RPKM value in round spermatids. Genes enriched in round spermatids are genes that are at least twofold upregulated compared to their RPKM value in pachytene spermatocytes. To visualize the distribution of RPKM values, Excel complementary module “Analysis ToolPak” was used to categorize genes according to their RPKM values (0–2, 2–4, 4–6, etc.).

### Annotation of the genome

Genome Decoration Page (http://www.ncbi.nlm.nih.gov/genome/tools/gdp/) was used to produce graphic representation of round spermatid-specific genes, *Speer* genes, H3K9me3 and RNA-Pol II genomic locations on chromosomes 3, 5, 6, 10, 14, 16, 18, X and Y.

### Immunofluorescence

Immunofluorescence experiments were performed on round spermatids following a protocol adapted from Barlow et al. [77] and previously described in [34]. Anti-H3K9me3 (Millipore) and anti-H3K27ac (active motif) were used at 1/200. Animal procedures were subjected to local ethical review (*Comite d’Ethique pour l’Experimentation Animale, Universite Paris Descartes*; registration number CEEA34.JC.114.12).

### Ensembl analysis

Ensembl BLAST Web site (http://www.ensembl.org/Multi/Tools/Blast) was used to search for genes related to takusan gene family. Using several gene accession IDs as queries, a total of 277 related genes (with >50% identity) were found; all mapped to chromosome 14. The analysis in which highly similar gene family members of the X and Y chromosome were treated as a single gene (Additional file 9) was performed as follows: Ensembl Biomart Web site (http://www.ensembl.org/biomart) was used to search for highly similar genes (with >80% identity) on the X and Y chromosomes. For the X chromosome, combination of the gene ID of identified members of multicopy gene families and the RNA-Seq data was used to analyze and collapse highly related genes into one single gene ID. For the Y chromosome, the multicopy gene-collapsed analysis was based on Soh et al. data and combined with the RNA-Seq data.

### Statistics

Analyses of chromatin marks coverage were performed by determining the 95% confidence interval (see Additional file 1). To compare the number of genes (based on their expression status) encoded by the sex chromosomes, chromosome 14 and representative autosomes, we performed Chi-square tests (see Additional files 5, 7 and 8). To compare distribution of RPKM values, Mann and Whitney tests were performed (see Additional files 6 and 9).

## Additional files

**Additional file 1.** Table showing individual chromosome coverage (reported to total chromosome length) of 9 histone PTM (i.e., H3K4me3, Kcr, H3K9ac, H4K8_hib, H4ac, K_acetylation, H3K9me3, H3K27ac, H3K27me3) and 5-hydroxymethylcytosine in round spermatids. Values are indicated for the sex chromosomes, chromosome 14 and representative autosomes. The mean coverage values, standard deviation (SD) and boundaries (maximum and minimum) values of the 95% confidence interval (CI) are reported underneath. Those values were calculated based on data collected from all chromosomes or from only autosomes (i.e., excluding values for the X and Y chromosomes). Values which are outside the 95% CI calculated for all chromosomes are represented in italic; values which are outside the 95% CI calculated for “only autosomes” are represented in bold and underscored.

**Additional file 2.** Graphic representation of the coverage (in base pair) of 9 histone PTM (i.e., H3K4me3, Kcr, H3K9ac, H4K8_hib, H4ac, K_acetylation, H3K9me3, H3K27ac, H3K27me3) and 5-hydroxymethylcytosine in round spermatids.

**Additional file 3.** Graphic representation of the ChIP-Seq profiles of H3K9me3, H3K27ac, H3K27me3, H4ac, H3K4me3 and Kcr over the sex chromosomes (X and Y) and two representative autosomes (chromosome 3 and chromosome 18) using IGV (Integrative Genomic Viewer). RefSeq genes are indicated underneath.

**Additional file 4.** Extended panel from Fig. 3. Immunofluorescence detection of H3K27ac (red) in round spermatid nuclei. DAPI (blue) was used to stain nuclei. The most DAPI-dense round region is the chromocenter (i.e., the constitutive pericentromeric heterochromatin) the less DAPI-dense structure adjacent to the chromocenter is the post-meiotic sex chromatin (PMSC) and is indicated by an arrow. Anti-H3K9me3 (in green) marks the chromocenter and the PMSC in wild type (WT) round spermatids. Two types of staining were observed: either a brighter signal co-localizing with the PMSC (top panel, *n*= 28/55), or a diffuse bright signal in the nucleus (middle panel, *n*=27/55). As control, round spermatids with a large deletion of the Y chromosome (MSYq-) were used (bottom panel). The vast majority of MSYq- round spermatids analyzed (59/65) did not display any enrichment of H3K27ac on the PMSC, indicating that in WT spermatids only the Y and not the X is enriched n H3K27ac.

**Additional file 5.** Tables presenting the results of Chi-square tests performed on the number of X- or Y-encoded expressed genes compared to autosomal expressed genes throughout spermatogenesis.

**Additional file 6.** Graphic representation of the mean rank values obtained with Mann and Whitney tests and tables presenting the results of Mann and Whitney tests performed on the RPKM values of X- and Y-encoded genes compared to that of autosomal genes in spermatogonia B (SB), pachytene spermatocytes (PS) and round spermatids (RS). P indicates the obtained p value (*, p < 0.05; **, p < 0.01; p < 0.001).

**Additional file 7.** Tables presenting the results of Chi-square tests performed on the number of pachytene (PS) repressed genes and PS repressed genes that are not reactivated in round spermatids (RS) for the X and Y chromosomes compared to representative autosomes.

**Additional file 8.** Tables presenting the results of Chi-square tests performed on the number of genes enriched in round spermatids (RS) and on the number of RS-specific genes for the X and Y chromosomes and chromosome 14 compared to representative autosomes.

**Additional file 9.** Analysis of single-copy genes and collapsed multicopy gene family of the sex chromosome.** a** Table presenting the number of X-encoded single-copy genes and multicopy gene family expressed throughout spermatogenesis, enriched in round spermatids (RS) or round spermatids (RS) specific. A Chi-square test has been performed on the number of genes expressed at each stage, genes enriched in RS or RS-specific genes for the X chromosome compared to representative autosomes. **b** Table presenting the number of Y-encoded single-copy genes and multicopy gene family expressed throughout spermatogenesis, enriched in round spermatids (RS) or round spermatids (RS) specific. A Chi-square test has been performed on the number of genes expressed at each stage, genes enriched in RS or RS-specific genes for the Y chromosome compared to representative autosomes. **c** Tables presenting the RPKM mean and RPKM sum of the Y-linked multicopy gene family expressed in round spermatids (RS).

**Additional file 10.** Graphic representation of the mean rank values obtained with Mann and Whitney tests and tables presenting the results of Mann and Whitney tests performed on the RPKM values of enriched genes in round spermatids (RS) and round spermatid (RS)-specific genes on the X and Y chromosomes compared to that of representative autosomes. P indicates the obtained p value (*, p < 0.05; **, p < 0.01; p < 0.001).

**Additional file 11.** List of the round spermatid-specific genes of the chromosome 14.

**Additional file 12.** Detailed protocol and information regarding the RNA-Seq and ChIP-Seq analysis.

**Additional file 13.** **a** RPKM threshold determination. Reads were mapped to Ensembl Genes (in blue) and to intergenic regions (in red). By comparing the expression levels of exons and intergenic regions, the intersection of the density plots was used to determine the threshold value to consider a gene as expressed (in our study= 0.22). **b** The true number of expressed genes in each bin (black) was estimated from the observed numbers for Ensembl genes (blue, same as **a**) by multiplication of the latter by the false discovery rate. This estimate was converted to cumulative amount, and the false negative rate was estimated as a function of expression level using the formula described in [48]. **c.** Bins were converted to cumulative amounts of genes expressed above the expression levels for genes (cum_genes; blue) and controls (cum_background; red). A false discovery rate fdr (green) was calculated at each expression level as described in [48].

## Abbreviations

ChIP Seq: chromatin immunoprecipitation followed by high-throughput sequencing
H3K4me3: trimethylation of lysine 4 on histone H3
H3K9ac: acetylation of lysine 9 on histone H3
H3K9me3: trimethylation of lysine 9 on histone H3
H3K27ac: acetylation of lysine 27 on histone H3
H3K27me3: trimethylation of lysine 27 on histone H3
H4ac: acetylation of histone H4
H4K8_ac: acetylation of lysine 8 on histone H4
H4K8_hib: 2-hydroxyisobutyrylation of lysine 8 on histone H4
K_acetylation: lysine acetylation
Kcr: lysine crotonylation
MSCI: meiotic sex chromosome inactivation
MSY: male-specific region of the Y chromosome
PMSC: post-meiotic sex chromatin, i.e., the X and Y chromatin in spermatids
PS: pachytene spermatocytes
PTM: posttranslational modifications
RPKM: reads per kilobase of transcript per million reads mapped
RNA Seq: high-throughput sequencing of RNA
RS: round spermatids
SB: spermatogonia B
TSS: transcription start site
uH2A: monoubiquitinated histone H2A
