# Supplementary material for: Expression and epigenomic landscape of the sex chromosomes in mouse post-meiotic male germ cells
Source: Epigenetics Chromatin. 2016 Oct 27;9:47. doi: 10.1186/s13072-016-0099-8 (PMC5081929; doi:10.1186/s13072-016-0099-8)
Supplement: Supplementary file 1 — Additional file 1. Table showing individual chromosome coverage (reported to total chromosome length) of 9 histone PTM (i.e., H3K4me3, Kcr, H3K9ac, H4K8_hib, H4ac, K_acetylation, H3K9me3, H3K27ac, H3K27me3) and 5-hydroxymethylcytosine in round spermatids. Values are indicated for the sex chromosomes, chromosome 14 and representative autosomes. The mean coverage values, standard deviation (SD) and boundaries (maximum and minimum) values of the 95% confidence interval (CI) are reported underneath. Those values were calculated based on data collected from all chromosomes or from only autosomes (i.e., excluding values for the X and Y chromosomes). Values which are outside the 95% CI calculated for all chromosomes are represented in italic; values which are outside the 95% CI calculated for “only autosomes” are represented in bold and underscored. [file 13072_2016_99_MOESM1_ESM.pdf]

coverage per chromosome (normalized to chromosome length)										
	H3K4me3	K_crotonylation	H3K9me3	H3K27me3	H3K9ac	H3K27ac	H4K8_hib	H4ac	K_acetylation	5hMC
chrX	0,023	0,023	0,0058	0,00068	0,031	0,006	0,00008	0,010	0,00009	0,0005
chrY	0,031	0,047	0,0170	0,00027	0,023	0,079	0,00011	0,007	0,00016	0,0005
chr3	0,026	0,034	0,0010	0,02800	0,033	0,002	0,00056	0,025	0,00045	0,0228
chr6	0,028	0,036	0,0004	0,02949	0,035	0,003	0,00072	0,028	0,00057	0,0324
chr14	0,040	0,046	0,0078	0,02884	0,043	0,006	0,00080	0,033	0,00039	0,0278
chr16	0,031	0,042	0,0009	0,03061	0,039	0,003	0,00079	0,031	0,00066	0,0325
chr18	0,029	0,037	0,0004	0,03299	0,035	0,002	0,00073	0,028	0,00039	0,0324
genome	0,033	0,042	0,0023	0,032	0,040	0,006	0,00081	0,031	0,00057	0,0380
All chromosomes										
mean	0,034	0,043	0,0024	0,033	0,040	0,007	0,0008	0,031	0,00058	0,039
SD	0,006	0,008	0,0039	0,012	0,007	0,017	0,0003	0,009	0,00022	0,018
95% CI max value	*0,045*	*0,058*	*0,0100*	*0,057*	*0,054*	*0,039*	*0,0014*	*0,049*	*0,00102*	*0,075*
95% CI min value	*0,023*	*0,028*	*-0,0053*	*0,009*	*0,027*	*-0,026*	*0,0002*	*0,013*	*0,00014*	*0,003*
Autosomes only										
mean	0,034	0,044	0,0014	0,036	0,042	0,003	0,0009	0,033	0,00063	0,043
SD	0,005	0,007	0,0018	0,006	0,006	0,001	0,0002	0,005	0,00018	0,014
95% CI max value	0,045	0,056	0,0049	0,048	0,053	0,005	0,0013	0,044	0,00097	0,070
95% CI min value	0,024	0,031	-0,0021	0,024	0,031	0,001	0,0005	0,023	0,00028	0,016

Additional file 1
