# Supplementary figures and images for: Expression and epigenomic landscape of the sex chromosomes in mouse post-meiotic male germ cells
Source: Epigenetics Chromatin. 2016 Oct 27;9:47. doi: 10.1186/s13072-016-0099-8 (PMC5081929; doi:10.1186/s13072-016-0099-8)

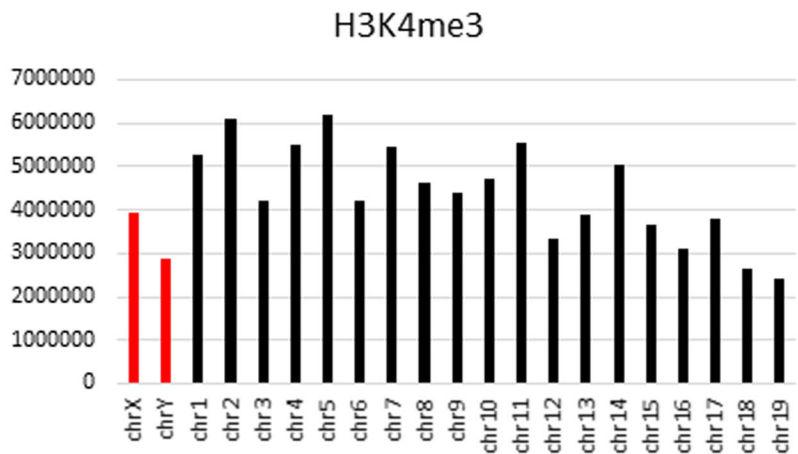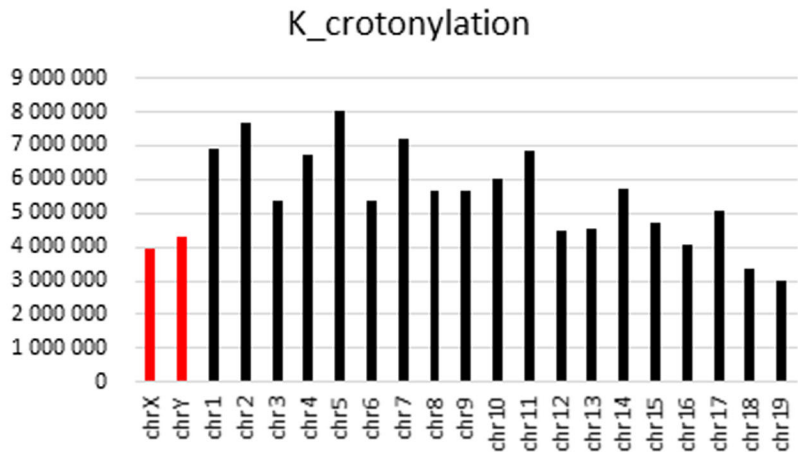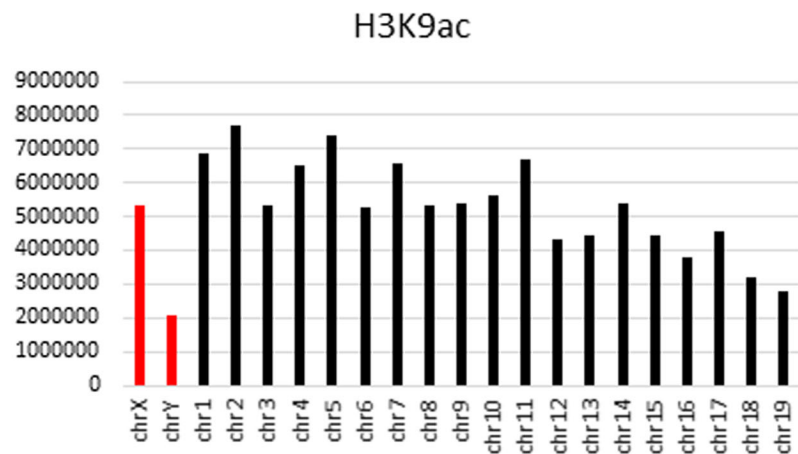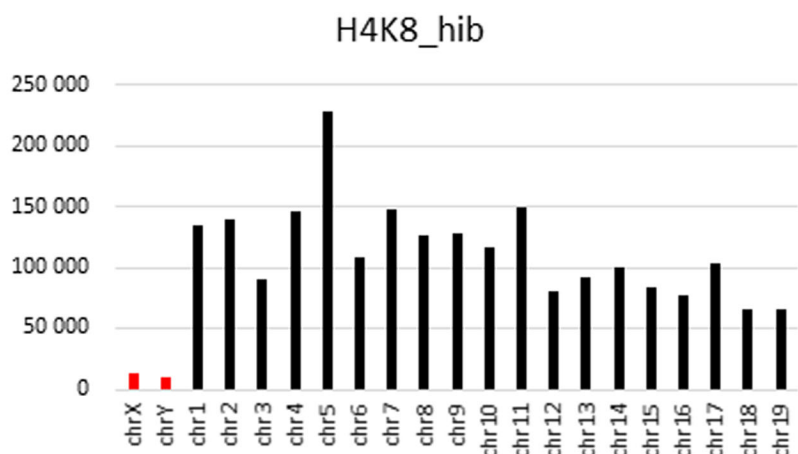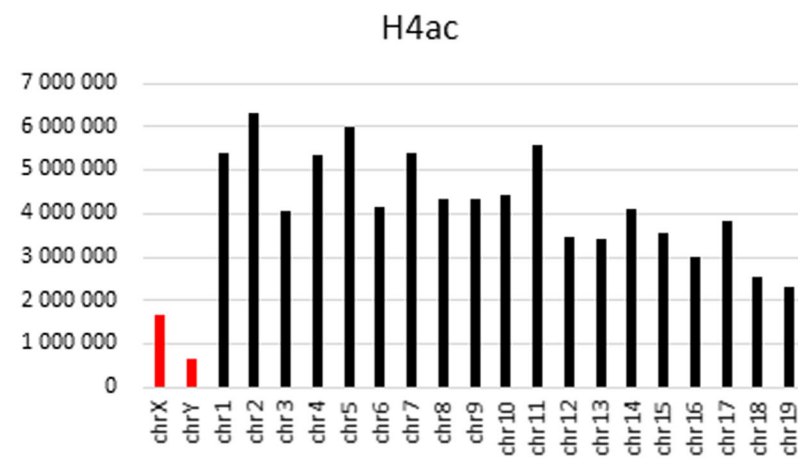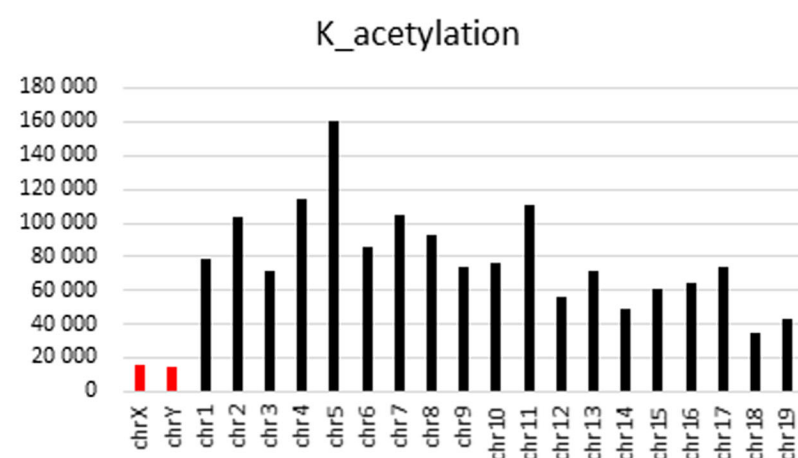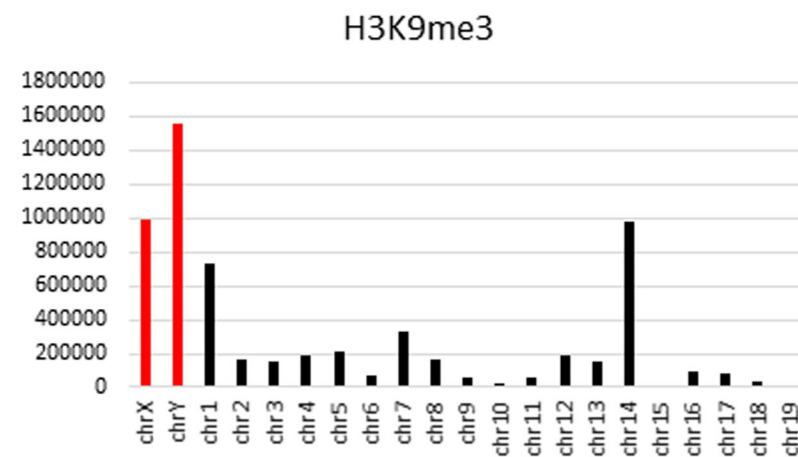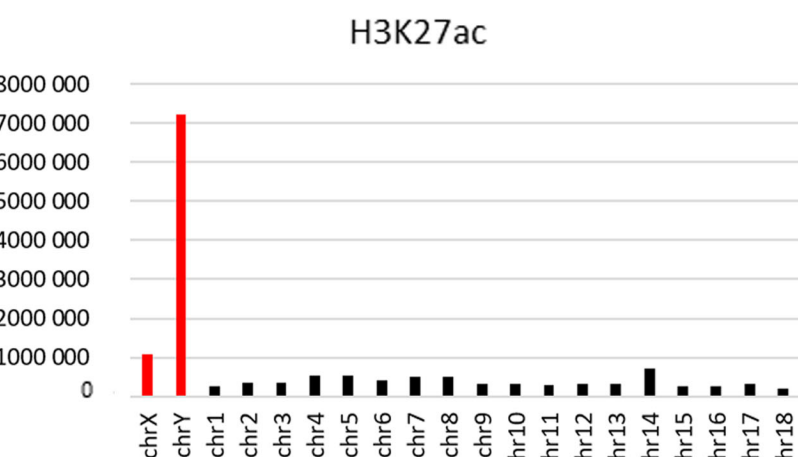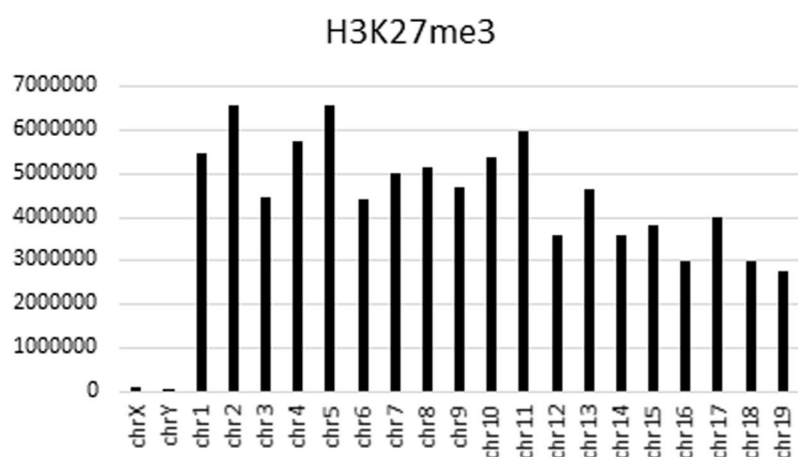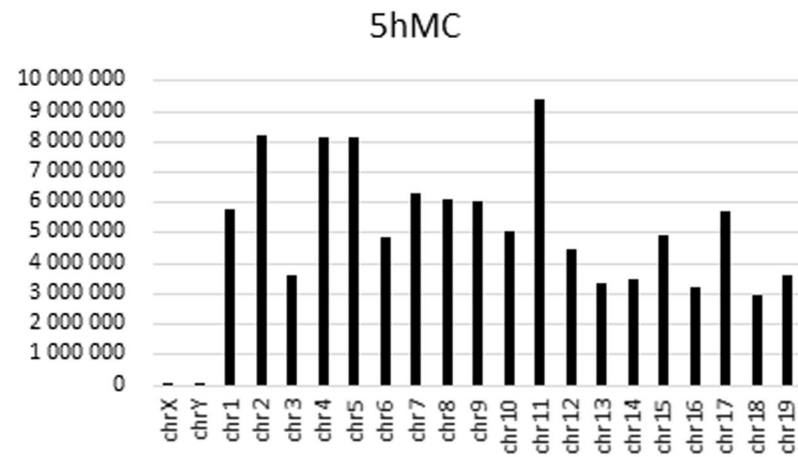

Supplement: Supplementary file 2 — Additional file 2. Graphic representation of the coverage (in base pair) of 9 histone PTM (i.e., H3K4me3, Kcr, H3K9ac, H4K8_hib, H4ac, K_acetylation, H3K9me3, H3K27ac, H3K27me3) and 5-hydroxymethylcytosine in round spermatids. [file 13072_2016_99_MOESM2_ESM.pdf]

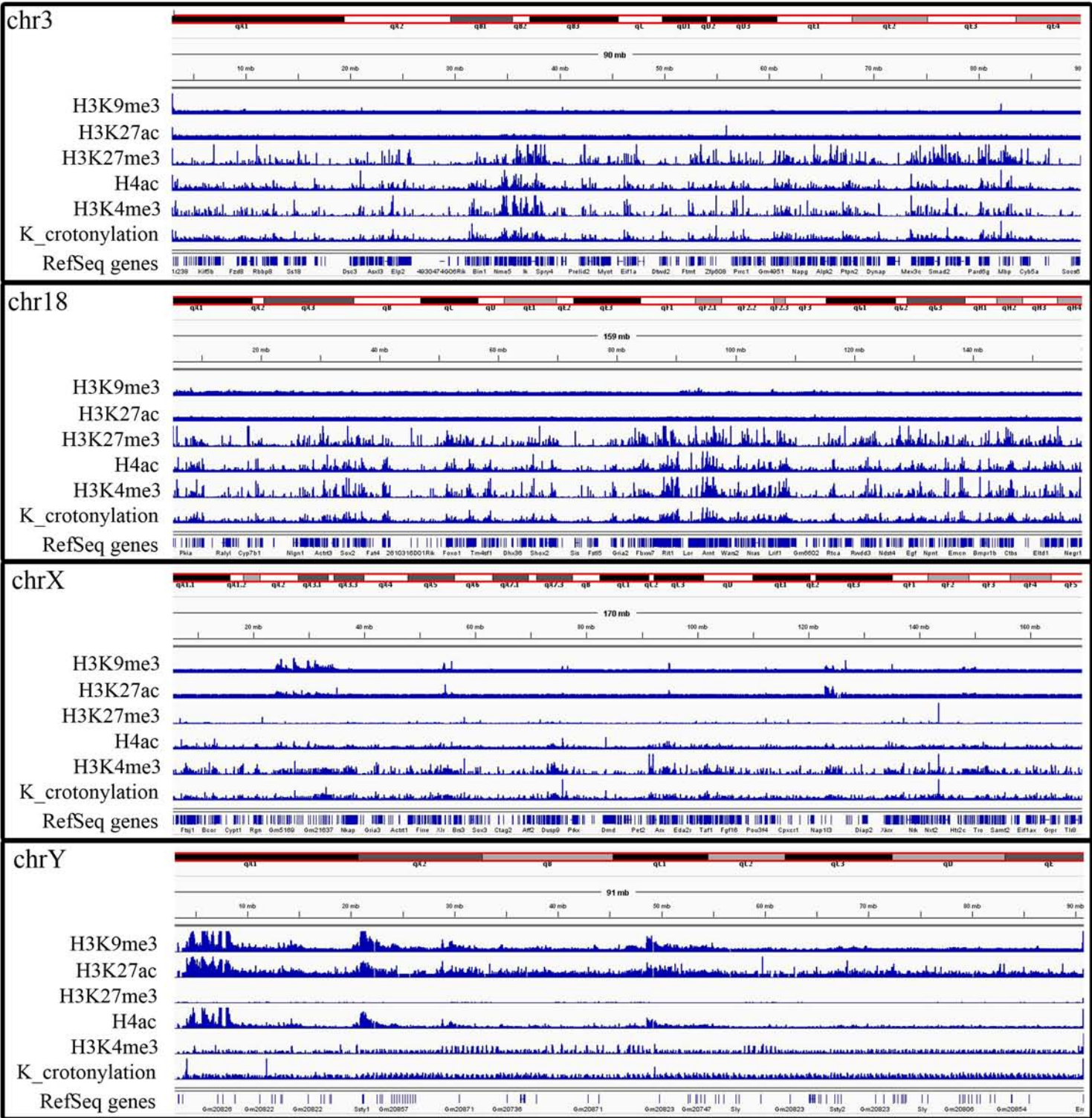

Supplement: Supplementary file 3 — Additional file 3. Graphic representation of the ChIP-Seq profiles of H3K9me3, H3K27ac, H3K27me3, H4ac, H3K4me3 and Kcr over the sex chromosomes (X and Y) and two representative autosomes (chromosome 3 and chromosome 18) using IGV (Integrative Genomic Viewer). RefSeq genes are indicated underneath. [file 13072_2016_99_MOESM3_ESM.pdf]

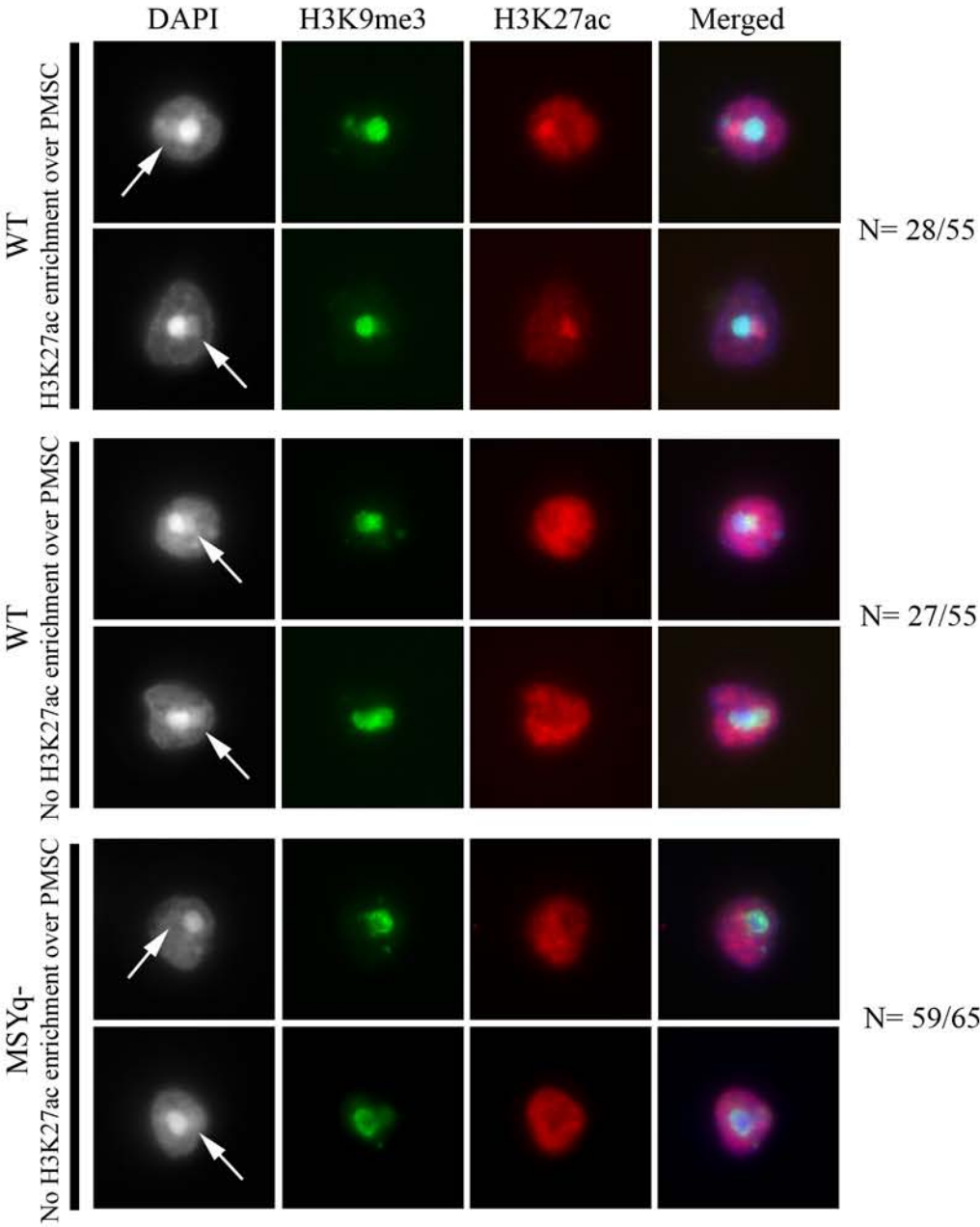

Supplement: Supplementary file 4 — Additional file 4. Extended panel from Fig. 3. Immunofluorescence detection of H3K27ac (red) in round spermatid nuclei. DAPI (blue) was used to stain nuclei. The most DAPI-dense round region is the chromocenter (i.e., the constitutive pericentromeric heterochromatin) the less DAPI-dense structure adjacent to the chromocenter is the post-meiotic sex chromatin (PMSC) and is indicated by an arrow. Anti-H3K9me3 (in green) marks the chromocenter and the PMSC in wild type (WT) round spermatids. Two types of staining were observed: either a brighter signal co-localizing with the PMSC (top panel, n= 28/55), or a diffuse bright signal in the nucleus (middle panel, n=27/55). As control, round spermatids with a large deletion of the Y chromosome (MSYq-) were used (bottom panel). The vast majority of MSYq- round spermatids analyzed (59/65) did not display any enrichment of H3K27ac on the PMSC, indicating that in WT spermatids only the Y and not the X is enriched n H3K27ac. [file 13072_2016_99_MOESM4_ESM.pdf]

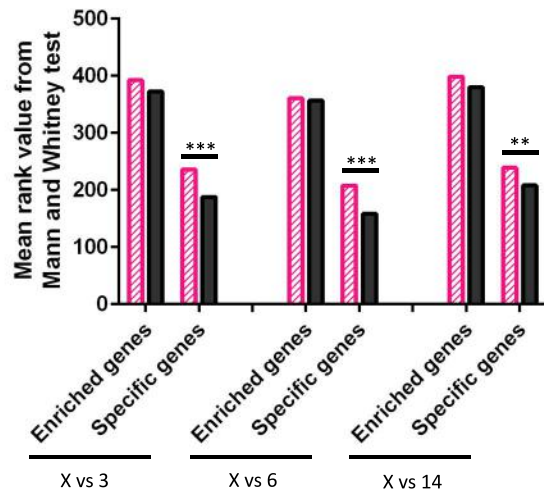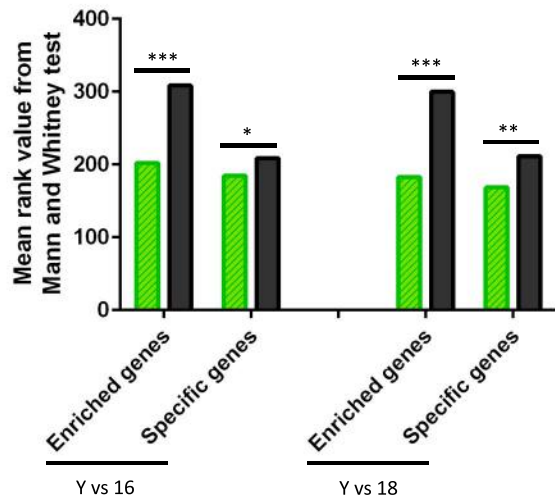

| Enriched in RS | p      |
|----------------|--------|
| X vs 3         | 0.1131 |
| X vs 6         | 0.4052 |
| X vs 14        | 0.1271 |

| Enriched in RS | p      |
|----------------|--------|
| Y vs 16        | <.0001 |
| Y vs 18        | <.0001 |

| RS specific | p      |
|-------------|--------|
| X vs 3      | <.0001 |
| X vs 6      | <.0001 |
| X vs 14     | 0.0059 |

| RS specific | p      |
|-------------|--------|
| Y vs 16     | 0.0401 |
| Y vs 18     | 0.0028 |

Supplement: Supplementary file 10 — Additional file 10. Graphic representation of the mean rank values obtained with Mann and Whitney tests and tables presenting the results of Mann and Whitney tests performed on the RPKM values of enriched genes in round spermatids (RS) and round spermatid (RS)-specific genes on the X and Y chromosomes compared to that of representative autosomes. P indicates the obtained p value (*, p < 0.05; **, p < 0.01; p < 0.001). [file 13072_2016_99_MOESM10_ESM.pdf]

**a** Estimation of noise for all samples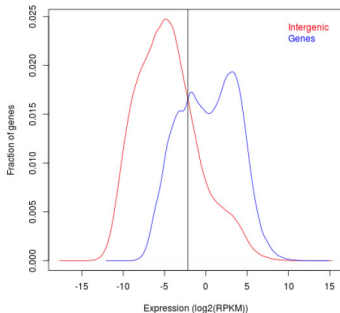

TRESHOLD:

Log2 (RPKM): -2,18

RPKM: 0,22

**b**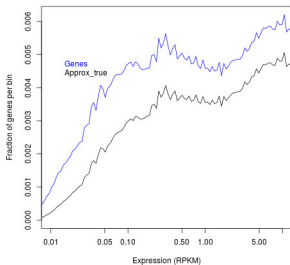**c**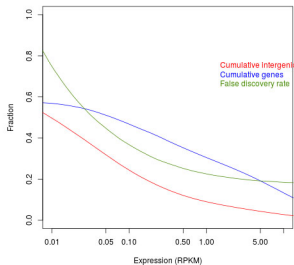

Supplement: Supplementary file 13 — Additional file 13. a RPKM threshold determination. Reads were mapped to Ensembl Genes (in blue) and to intergenic regions (in red). By comparing the expression levels of exons and intergenic regions, the intersection of the density plots was used to determine the threshold value to consider a gene as expressed (in our study= 0.22). b The true number of expressed genes in each bin (black) was estimated from the observed numbers for Ensembl genes (blue, same as a) by multiplication of the latter by the false discovery rate. This estimate was converted to cumulative amount, and the false negative rate was estimated as a function of expression level using the formula described in [48]. c. Bins were converted to cumulative amounts of genes expressed above the expression levels for genes (cum_genes; blue) and controls (cum_background; red). A false discovery rate fdr (green) was calculated at each expression level as described in [48]. [file 13072_2016_99_MOESM13_ESM.pdf]
