# Supplementary material for: Expression and epigenomic landscape of the sex chromosomes in mouse post-meiotic male germ cells
Source: Epigenetics Chromatin. 2016 Oct 27;9:47. doi: 10.1186/s13072-016-0099-8 (PMC5081929; doi:10.1186/s13072-016-0099-8)
Supplement: Supplementary file 5 — Additional file 5. Tables presenting the results of Chi-square tests performed on the number of X- or Y-encoded expressed genes compared to autosomal expressed genes throughout spermatogenesis. [file 13072_2016_99_MOESM5_ESM.pdf]

Additional file 5: Analysis of expressed genes for X and Y chromosomes throughout spermatogenesis

p value of χ^2test for X chromosome *vs* autosomes	Y	3	6	14
For spermatognia B cells	1.83E-88	4.67E-01	6.06E-03	1.74E-03
For pachytene stage cells	6.69E-76	2.13E-06	7.89E-07	9.74E-02
For round spermatids cells	3.95E-08	7.20E-01	2.40E-01	4.75E-05
For elongating cells	1.59E-04	2.86E-01	5.02E-01	9.58E-04

p value of χ^2test for X chromosome stages	
For SB *vs* PS	1.65E-03
For PS *vs* RS	1.10E-17
For SB *vs* RS	4.80E-08
For RS *vs* ES	5.64E-02

p value of χ^2test for Y chromosome *vs* autosomes	16	18
For spermatognia B cells	1.29E-87	6.02E-86
For pachytene stage cells	3.02E-90	7.03E-87
For round spermatids cells	1.30E-03	8.88E-06
For elongating cells	4.14E-02	7.43E-03

p value of χ^2test for Y chromosome stages	
For SB *vs* PS	3.96E-01
For PS *vs* RS	9.90E-111
For SB *vs* RS	2.49E-106
For RS *vs* ES	7.91E-03
