# Supplementary material for: Expression and epigenomic landscape of the sex chromosomes in mouse post-meiotic male germ cells
Source: Epigenetics Chromatin. 2016 Oct 27;9:47. doi: 10.1186/s13072-016-0099-8 (PMC5081929; doi:10.1186/s13072-016-0099-8)
Supplement: Supplementary file 6 — Additional file 6. Graphic representation of the mean rank values obtained with Mann and Whitney tests and tables presenting the results of Mann and Whitney tests performed on the RPKM values of X- and Y-encoded genes compared to that of autosomal genes in spermatogonia B (SB), pachytene spermatocytes (PS) and round spermatids (RS). P indicates the obtained p value (*, p < 0.05; **, p < 0.01; p < 0.001). [file 13072_2016_99_MOESM6_ESM.pdf]

Additional file 6: Mann and Whitney test on the RPKM values of X and Y chromosomes throughout spermatogenesis

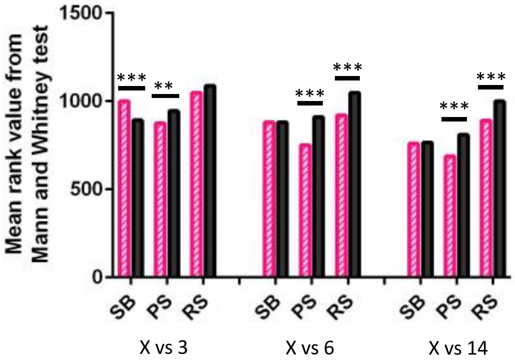


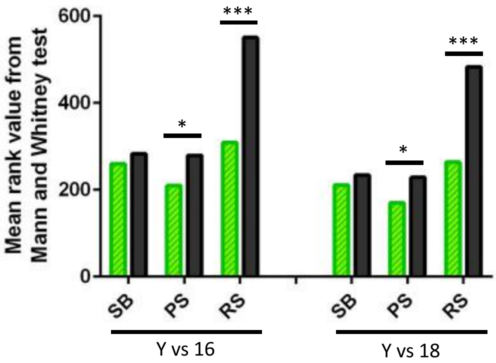


| SB | p |
|---|---|
| X vs 3 | <.0001 |
| X vs 6 | 0.5 |
| X vs 14 | 0.3974 |

| PS | p |
|---|---|
| X vs 3 | 0.0032 |
| X vs 6 | <.0001 |
| X vs 14 | <.0001 |

| RS | p |
|---|---|
| X vs 3 | 0.0643 |
| X vs 6 | <.0001 |
| X vs 14 | <.0001 |

| SB | p |
|---|---|
| 16 vs Y | 0.2297 |
| 18 vs Y | 0.1841 |

| PS | p |
|---|---|
| 16 vs Y | 0.0188 |
| 18 vs Y | 0.0162 |

| RS | p |
|---|---|
| 16 vs Y | <.0001 |
| 18 vs Y | <.0001 |
