# Supplementary material for: Expression and epigenomic landscape of the sex chromosomes in mouse post-meiotic male germ cells
Source: Epigenetics Chromatin. 2016 Oct 27;9:47. doi: 10.1186/s13072-016-0099-8 (PMC5081929; doi:10.1186/s13072-016-0099-8)
Supplement: Supplementary file 7 — Additional file 7. Tables presenting the results of Chi-square tests performed on the number of pachytene (PS) repressed genes and PS repressed genes that are not reactivated in round spermatids (RS) for the X and Y chromosomes compared to representative autosomes. [file 13072_2016_99_MOESM7_ESM.pdf]

Additional file 7: χ^2 test on the number of PS repressed genes and PS repressed genes that are not reactivated in RS for X and Y chromosomes

**p value of χ^2test for X chromosomes *vs* autosomes**	**Y**	**3**	**6**	**14**
**For the proportion of pachytene repressed genes**	7.62E-02	1.22E-50	2.25E-46	2.15E-33
**For the proportion PS repressed genes that are not reactivated in RS**	4.20E-01	5.64E-01	5.83E-02	6.55E-09

**p value of χ^2test for Y chromosomes *vs* autosomes…**	**16**	**18**
**For the proportion of pachytene repressed genes**	2.24E-07	1.20E-06
**For the proportion PS repressed genes that are not reactivated in RS**	7.46E-02	2.58E-01
