# Supplementary material for: Expression and epigenomic landscape of the sex chromosomes in mouse post-meiotic male germ cells
Source: Epigenetics Chromatin. 2016 Oct 27;9:47. doi: 10.1186/s13072-016-0099-8 (PMC5081929; doi:10.1186/s13072-016-0099-8)
Supplement: Supplementary file 8 — Additional file 8. Tables presenting the results of Chi-square tests performed on the number of genes enriched in round spermatids (RS) and on the number of RS-specific genes for the X and Y chromosomes and chromosome 14 compared to representative autosomes. [file 13072_2016_99_MOESM8_ESM.pdf]

Additional file 8: χ^2 test on the number of genes enriched in RS and RS specific genes for X and Y chromosomes and chromosome 14

**p value of χ^2test for X chromosome *vs* autosomes**	**Y**	**3**	**6**	**14**
**For the proportion of genes enriched in RS**	6.89E-75	2.15E-03	2.24E-02	2.20E-03
**For the proportion of RS specific genes**	3.91E-118	6.26E-06	4.12E-08	7.28E-01

**p value of χ^2test for Y chromosome *vs* autosomes**	**16**	**18**
**For the proportion of genes enriched in RS**	7.40E-78	9.09E-75
**For the proportion of RS specific genes**	1.00E-124	5.76E-119

**p value of χ^2test for chromosome 14**	**10**
**For the proportion of genes enriched in RS**	1.94E-12
**For the proportion of RS specific genes**	1.02E-12
