# Supplementary material for: Expression and epigenomic landscape of the sex chromosomes in mouse post-meiotic male germ cells
Source: Epigenetics Chromatin. 2016 Oct 27;9:47. doi: 10.1186/s13072-016-0099-8 (PMC5081929; doi:10.1186/s13072-016-0099-8)
Supplement: Supplementary file 9 — Additional file 9. Analysis of single-copy genes and collapsed multicopy gene family of the sex chromosome. a Table presenting the number of X-encoded single-copy genes and multicopy gene family expressed throughout spermatogenesis, enriched in round spermatids (RS) or round spermatids (RS) specific. A Chi-square test has been performed on the number of genes expressed at each stage, genes enriched in RS or RS-specific genes for the X chromosome compared to representative autosomes. b Table presenting the number of Y-encoded single-copy genes and multicopy gene family expressed throughout spermatogenesis, enriched in round spermatids (RS) or round spermatids (RS) specific. A Chi-square test has been performed on the number of genes expressed at each stage, genes enriched in RS or RS-specific genes for the Y chromosome compared to representative autosomes. c Tables presenting the RPKM mean and RPKM sum of the Y-linked multicopy gene family expressed in round spermatids (RS). [file 13072_2016_99_MOESM9_ESM.pdf]

Additional file 9: Analysis of single copy genes and collapsed multicopy gene family of the sex chromosomes.

a.

**X-linked genes**	**Single copy genes**	**Multicopy gene family**	**% of expressed genes**
Expressed at some point during spermatogenesis	*1140*	*50*	*49,17*
**Expressed in spermatogonia B cells**	806	32	70,42
**Expressed in pachytene stage cells**	726	35	63,95
**Expressed in round spermatids cells**	855	48	75,88
**Enriched in RS**	308	48	29,92
**RS specific**	176	21	16,55

**p value of χ^2 test for X chromosome *vs* autosomes**	**3**	**6**
**For spermatognia B cells**	3,83E-01	2,42E-01
**For pachytene stage cells**	6,16E-04	2,28E-04
**For round spermatids cells**	2,78E-01	6,44E-02
**For genes enriched in RS**	7,11E-03	5,19E-02
**For RS specific genes**	4,07E-03	8,40E-05

b.

**Y-linked genes**	**Single copy genes**	**Multicopy gene family**	**% of expressed genes**
Expressed at some point during spermatogenesis	*12*	*4*	*94,12*
**Expressed in spermatogonia B cells**	8	1	56,25
**Expressed in pachytene stage cells**	8	1	56,25
**Expressed in round spermatids cells**	10	4	87,50
**Enriched in RS**	3	3	37,50
**RS specific**	3	3	37,50

**p value of χ^2 test for Y chromosome *vs* autosomes**	**3**	**6**
**For spermatognia B cells**	1,58E-01	1,05E-01
**For pachytene stage cells**	1,79E-01	1,40E-01
**For round spermatids cells**	6,27E-01	4,14E-01
**For genes enriched in RS**	2,37E-01	1,81E-01
**For RS specific genes**	8,22E-04	6,86E-05

c.

**Y-linked genes multicopy gene famiilly**	**RPKM mean in RS**	**RPKM sum in RS**
Sly (32 out of 126 copies)	1,16	37,09
Ssty1/2 (28 out of 306 copies)	0,92	25,91
Rbmy (9 out of 30 copies)	3,32	29,92
Srsy (zero out of 197 copies)		
