# Supplementary material for: Expression and epigenomic landscape of the sex chromosomes in mouse post-meiotic male germ cells
Source: Epigenetics Chromatin. 2016 Oct 27;9:47. doi: 10.1186/s13072-016-0099-8 (PMC5081929; doi:10.1186/s13072-016-0099-8)
Supplement: Supplementary file 11 — Additional file 11. List of the round spermatid-specific genes of the chromosome 14. [file 13072_2016_99_MOESM11_ESM.pdf]

Additional file 11: List of the round spermatid specific genes of the chromosome 14

Gm2888	Gm8024	Gm3072	Gm25517	Gm9780
Gm10340	Gm17124	Gm3676	4930444M15Rik	Slmapos2
Gm5795	Gm5929	Gm8065	1700108F19Rik	mmu-mir-6947
Gm10413	Gm3008	Gm8068	Zfp957	1700087M22Rik
Gm3012	Gm10377	Gm7929	Gm25115	Colq
Gm3002	Gm21977	Gm7980	Cdhr1	Slc18a3
Gm3095	Gm10376	Gm6401	1700120O09Rik	3425401B19Rik
Gm3099	Gm8094	Gm3543	4930596D02Rik	1810011H11Rik
Gm8108	Gm8122	Gm2930	Gm7853	Fam170b
Gm3127	Gm8127	Gm7991	4930474N05Rik	Gm26228
Gm3138	1700001F09Rik	1700091H14Rik	Pnma2	Gm5460
Gm3159	Gm8138	Gm7951	4930578I07Rik	Gm7945
Gm3182	Gm17654	Gm17027	Gm27647	Gm6482
Gm3030	Gm10375	Gm3573	Antxrl	Gm7954
Gm8159	Gm8046	Gm17026	A630023A22Rik	Gm3486
Gm7876	Gm8165	Gm9611	Gm17116	Gm7970
Gm9602	Gm16506	Gm8005	Chrna2	4930438E09Rik
Gm3269	Gm8180	Gm8020	Gm37034	Gm10860
Gm3264	Ear-ps8	Pcdh9	Gzmd	Gm31748
Gm3278	Gm16545	4921530L21Rik	Gzmg	Gm16549
Gm3298	Gm3327	Gm15515	Gzmn	4930564B18Rik
4930555G01Rik	Gm8212	4930517O19Rik	Gjb6	Gm3715
Gm3339	Gm8220	Slain1os	4930563I02Rik	Gm3727
Gm3542	Gm8229	4930449E01Rik	Amer2	Gm3752
Gm8246	Gm8232	Mir6390	Arl11	Gm3558
Gm3187	BC061237	1700128A07Rik	1700109G14Rik	Dnase1l3
Gm9603	Gm8247	Slitrk1	Gm27928	Fhitos
Gm3424	Gm8256	4930505G20Rik	Blk	Gm3848
Gm17158	Gm21754	Gm26791	Gm21430	Gm8582
Gm3242	Gm16976	1700044C05Rik	4930578I06Rik	Gm9637
Gm8206	Gm10101	Gm27198	Prss55	Dusp13
Gm26991	1700011H14Rik	1700006F04Rik	Prss51	Gm17175
Gm21560	Olfr731	Hs6st3	4930471C04Rik	Gm17174
Gm16440	Olfr732	Gm17613	Prss52	Gm17078
Gm3594	Olfr733	Zic5	E330034G19Rik	Gm4181
Gm8356	Gm21718	2610528A11Rik	4930542C16Rik	Gm17079
Gm3642	Gm3719	Nrg3os	4931403M11Rik	Gm5800
Gm6356	Vmn2r88	Gm20642	4930428N03Rik	AY358078
Gm6676	Vmn2r-ps111	1700109I08Rik	1700054O19Rik	Olfr1508
Gm16434	Gm7247	Sh2d4b	Anxa11os	Olfr1507
				Mhrt
