# Supplementary material for: Expression and epigenomic landscape of the sex chromosomes in mouse post-meiotic male germ cells
Source: Epigenetics Chromatin. 2016 Oct 27;9:47. doi: 10.1186/s13072-016-0099-8 (PMC5081929; doi:10.1186/s13072-016-0099-8)
Supplement: Supplementary file 12 — Additional file 12. Detailed protocol and information regarding the RNA-Seq and ChIP-Seq analysis. [file 13072_2016_99_MOESM12_ESM.pdf]

Additional file 12 - 1/2

RNA-Seq analyses datasets from [47]

Mapping with STAR 2.4.2a [75] on Mouse Genome version GRCm38 mm10.

Command line: *STAR --outSAMstandField intronMotif --outFilterMismatchNmax 2 --outFilterMultmapNmax 10 --genomeDir $Starindex --readFilesIn $File1 --runThreadN $threads --outSAMunmapped Within --outSAMtype BAM SortedByCoordinate --outStd BAM_SortedByCoordinate --genomeLoad NoSharedMemory -- chimSegmentMin 15 --limitBAMsortRAM $starlimitRAM*

FPKM count using FeatureCounts (Subread package version 1.4.6-p5) [76]

Command line: *featureCounts -a Mus_musculus.GRCm38.81.gtf -o counts.txt -A chrnames.txt -t exon -g gene_id --primary -T 30 GSM860181.bam GSM860182.bam GSM860183.bam GSM860184.bam GSM860185.bam GSM860186.bam GSM860187.bam GSM860188.bam*

Sample	cell type	Number of reads	Uniquely mapped reads number	Number of reads mapped to multiple loci	Number of reads mapped to too many loci	% of unmapped reads	% of mapped reads
GSM860181	priSG-A	46 531 667	36 259 756	4 731 871	217 517	0,5%	99,5%
GSM860182	SG-A	40 616 823	32 616 138	3 698 384	176 306	0,4%	99,6%
GSM860183	SG-B	30 856 488	24 464 384	2 801 768	125 578	0,4%	99,6%
GSM860184	lepSC	39 869 596	31 960 883	3 334 547	144 071	0,4%	99,6%
GSM860185	pacSC	32 436 551	26 541 732	2 453 985	84 493	0,3%	99,7%
GSM860186	rST	38 101 736	31 668 437	2 022 790	101 116	0,3%	99,7%
GSM860187	eST	30 363 718	25 706 211	1 562 476	63 762	0,2%	99,8%
GSM860188	SE	36 991 035	30 380 459	3 444 961	158 240	0,4%	99,6%

Additional file 12 - 2/2

ChIP-Seq analyses

Mapping with Heng Li Burros-Wheeler aligner BWA [72] version 0.7.5a-r405 with "mem" command against Mouse Genome version GRCm38 mm10

Command line: *bwa mem -t 6 ref_mouse.fa sample.fastq*

Peak calling using MACS [73] v1.4

Command line: *macs14 -t chip_sample.bam -c input_sample.bam -f BAM -g mm -n MACS_sample_mem*

Sample ID (NCBI)	Mark	Number of reads	Number of peaks	% of mapped reads	Reference
GSM1046840-GSM1046841	H3K4me3 (duplicate)	40783145-225974316	32058-40767	78,92-89,84%	[71]
GSM1046842-GSM1046843	H3K27me3 (duplicate)	33620246-225140169	30472-38805	83,11%-93,42%	[71]
GSM810678	K_crotonylation	133 523 062	40 211	80,67%	[39]
GSM810677	K_acetylation	51 481 906	7 099	84,08%	[39]
GSM810676	Corresponding Input file	43 928 317	N/A	82,34%	[39]
Courtesy of S. Rousseaux	H4K8_hib	26 649 536	14 908	91,70%	[41]
Courtesy of S. Rousseaux	Corresponding Input file	31 177 064	N/A	95,25%	[41]
GSM1519003	H3K9me3	84 339 867	11 659	90,34%	[57]
GSM1519004	H3K9ac	67 856 707	47 333	92,04%	[57]
GSM1519009	H4ac	70 049 830	44 966	93,37%	[57]
GSM1519010	Corresponding Input file	54 990 464	N/A	94,38%	[57]
SRR948811/GSM1202715	H3K27ac	245 489 610	123 434	64,51%	[70]
SRR948814/GSM1202718	5hMC	52 517 270	125 719	96,79%	[70]
SRR948825/GSM1202725	Corresponding Input file	153 674 411	N/A	81,15%	[70]
